# Wearable sensor-based quantitative gait analysis in Parkinson’s disease patients with different motor subtypes

**DOI:** 10.1038/s41746-024-01163-z

**Published:** 2024-06-26

**Authors:** Weishan Zhang, Yun Ling, Zhonglue Chen, Kang Ren, Shengdi Chen, Pei Huang, Yuyan Tan

**Affiliations:** 1 Department of Neurology and Institute of Neurology, Ruijin Hospital, Shanghai Jiao Tong University School of Medicine, Shanghai, China; 2 GYENNO SCIENCE Co., Ltd. Department of Research, Shenzhen, Guangdong China; 3 HUST-GYENNO CNS Intelligent Digital Medicine Technology Center, Wuhan, China

**Keywords:** Parkinson's disease, Translational research, Diagnostic markers

## Abstract

Gait impairments are among the most common and disabling symptoms of Parkinson’s disease and worsen as the disease progresses. Early detection and diagnosis of subtype-specific gait deficits, as well as progression monitoring, can help to implement effective and preventive personalized treatment for PD patients. Yet, the gait features have not been fully studied in PD and its motor subtypes. To characterize comprehensive and objective gait alterations and to identify the potential gait biomarkers for early diagnosis, subtype differentiation, and disease severity monitoring. We analyzed gait parameters related to upper/lower limbs, trunk and lumbar, and postural transitions from 24 tremor-dominant (TD) and 20 postural instability gait difficulty (PIGD) dominant PD patients who were in early stage and 39 matched healthy controls (HC) during the Timed Up and Go test using wearable sensors. Results show: (1) Both TD and PIGD groups showed restricted backswing range in bilateral lower extremities and more affected side (MAS) arm, reduced trunk and lumbar rotation range in the coronal plane, and low turning efficiency. The receiver operating characteristic (ROC) analysis revealed these objective gait features had high discriminative value in distinguishing both PD subtypes from the HC with the area under the curve (AUC) values of 0.7~0.9 (*p* < 0.01). (2) Subtle but measurable gait differences existed between TD and PIGD patients before the onset of clinically apparent gait impairment. (3) Specific gait parameters were significantly associated with disease severity in TD and PIGD subtypes. Objective gait biomarkers based on wearable sensors may facilitate timely and personalized gait treatments in PD subtypes through early diagnosis, subtype differentiation, and disease severity monitoring.

## Introduction

Parkinson’s disease (PD) is a progressive neurological disorder manifested by a broad spectrum of motor and non-motor symptoms^1^. Currently, PD is clinically defined as the presence of bradykinesia combined with varying degrees of rest tremor, rigidity, or gait disorders and postural instability^2,3^. The variability of motor symptoms forms the basis of classification of motor subtypes, which are typically classified as tremor-dominant (TD), postural instability and gait difficulty (PIGD) dominant, or indeterminate (IND) subtype-based on the sub-scores of the Movement Disorders Society-Unified Parkinson’s Disease Rating Scale (MDS-UPDRS)^4^. The clinical features and prognosis of patients with PD vary according to the motor subtypes^5–10^. Several longitudinal studies have suggested that the PIGD subtype is more aggressive compared with the TD subtype, in terms of disease survival, risk of dementia, depression, and quality of life^8,11^. Gait impairments are among the most common and disabling symptoms of PD and worsen as the disease progresses^12,13^. It remains very difficult to satisfactorily alleviate gait disturbances. At present, three main clinical strategies available to treat PD patients consist of pharmaceutical, surgical, and adapted physical activity or physiotherapy interventions. Although the available medications improve certain aspects of walking, most gait symptoms are less responsive to medication^14^. In addition, the gold-standard dopaminergic treatments also create multiple challenges that can further impair gait^15^. Unlike tremor, gait impairment in PD responds insufficiently to surgical treatment^16^. An increasing number of studies have explored the effects of exercise training for PD patients and proved that physical activity, such as balance training, walking exercises, muscle strengthening, and stretching exercises could improve balance and gait ability^17,18^.

However, PD patients with different subtypes exert diverse impairment in gait patterns due to the differences in biometric characteristics and motor symptoms, identifying gait features that are associated with specific motor subtype will help clinicians to anticipate gait impairment and treat or intervene them promptly, especially in early phase of the disease. Hence, we believe the exploration of the subtype-based gait features in early PD patients may facilitate the development of personalized treatment for gait impairments in PD. Moreover, as the PIGD subtype has been associated with faster clinical progression, whereas TD subtype is associated with a better prognosis^8,10^, the closer monitoring and earlier implementation of appropriate intervention for motor subtype is clinically meaningful, which also highlights the need for biomarkers that can accurately predict disease progression according to motor subtypes. Therefore, early detection and diagnosis of subtype-specific gait deficits, as well as disease progression monitor, can help to implement effective and preventive personalized treatment for PD patients.

Although, previous studies have investigated the different gait features between TD and PIGD subtypes^9,19–23^, most studies mainly focused on lower body-related features, such as step length, step velocity, or stride regularity^9,20–23^. At the early stage of PD, slow gait speed and short step length are always first observed, however, these gait impairments are not PD-specific signs, as they are age-related and can be induced by many other diseases^24^, indicating that the conventional gait parameters may be difficult for discriminating subtle gait changes. In addition, optimal evaluation and treatment of gait alterations in PD patients demands an understanding of the multiple mechanisms and factors that contribute to these problems. However, gait is not routinely assessed quantitatively but is described in general terms that are not sensitive to changes ensuing with disease progression^24^. Supported by advanced sensing technologies, wearable sensors can be used for supporting PD diagnosis, differential diagnosis between various neurological disorders, detection or prediction of PD symptoms, and estimation of their severity, such as tremor, freezing of gait (FoG), and motor fluctuations^25^. Particularly, advances in small, body-worn, inertial sensors have made it possible to develop objective measures of multifaceted gait features, including the axial, upper, and lower body-related parameters^26,27^, aiming to provide a comprehensive profile of gait features and identify subtle gait alterations in the early stage of PD by quantifying multiple gait features^28^. Moreover, unlike the other multifaceted gait analysis based on the motion capture system^29–31^, the wearable sensor-based gait analysis does not involve expensive, highly technical, non-portable equipment which is limited to be applied in advanced biokinetics laboratory and makes it possible to develop quick, objective measures of balance and gait impairments in the clinic for research trials and clinical practice^26,27,32,33^.

Previous studies have proved that the combination of wearable sensors with the Timed Up and Go (TUG) test, a widely used clinical assessment of functional mobility^34–39^, was sensitive to assess mobility in PD and could potentially detect the disease progression^26,40^. As the TUG tests several different mobility skills. These include sit-to-stand and stand-to-sit chair transitions, turning, straight-ahead gait, balance control, and the ability to sequence tasks^41–43^, which all could be affected in PD. Therefore, the TUG test has been deemed a highly suitable examination for assessing motor symptoms in PD^44^. In recent years, increasing interest has been focused on wearable sensors-based objective gait measurement in PD patients^26,40,45^. However, most gait features obtained from single inertial sensor located in lower back or two sensors fixed to bilateral lower limbs, so limited data were available on gait features related to upper limbs and trunk. Moreover, most studies have been focused on gait alterations in advanced PD, in which gait and postural abnormalities are more evident in clinical observation, whereas less attention has been paid to gait features in the early phase of the disease. In addition, the relationships between the gait features and disease severity in PD subtypes have not been fully described.

**Fig. 1:**
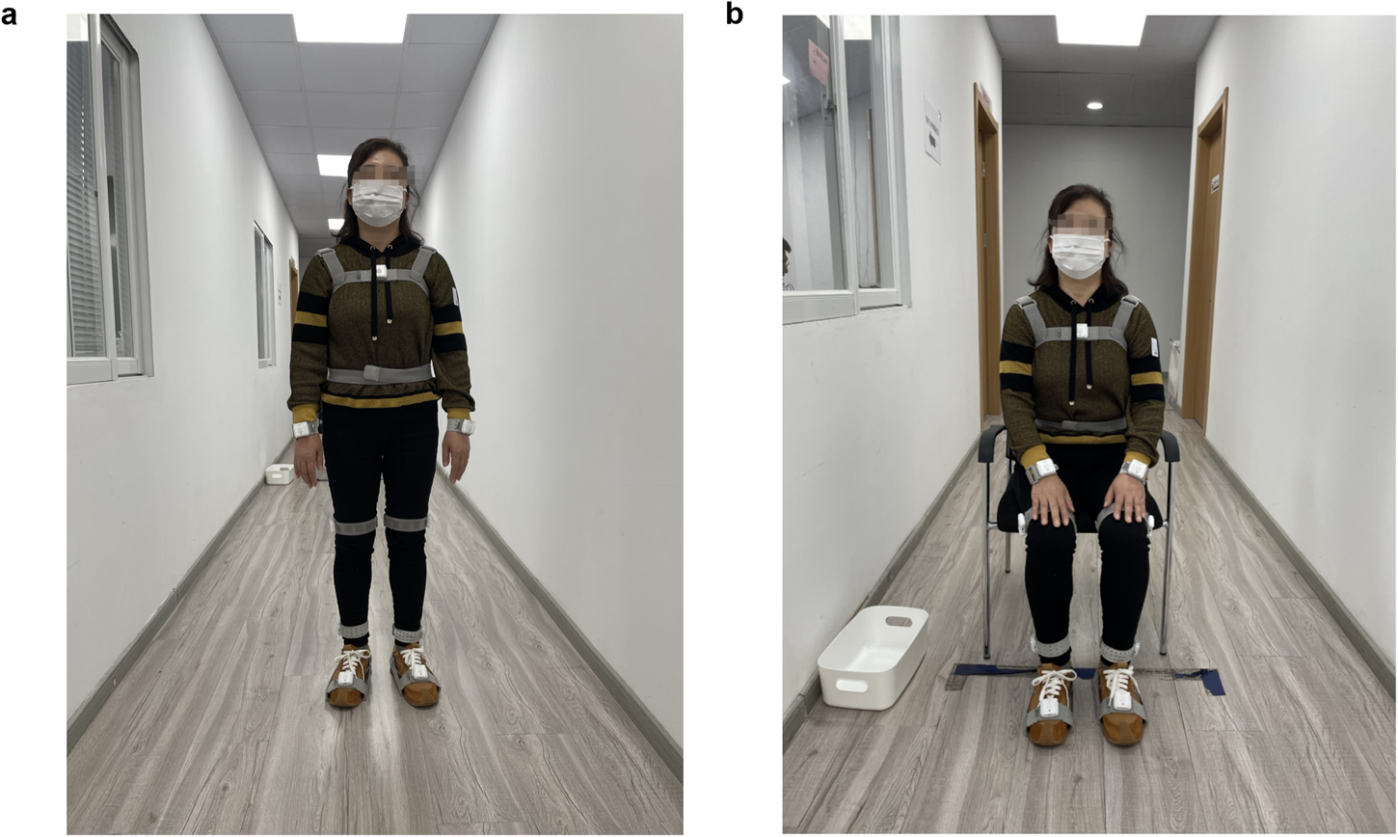
Ten body-fixed sensors are attached to each subject’s chest, lower back, and bilateral wrists, thighs, ankles, and feet. Pictures are shown as standing (**a**) and sitting (**b**) conditions.

**Fig. 2:**
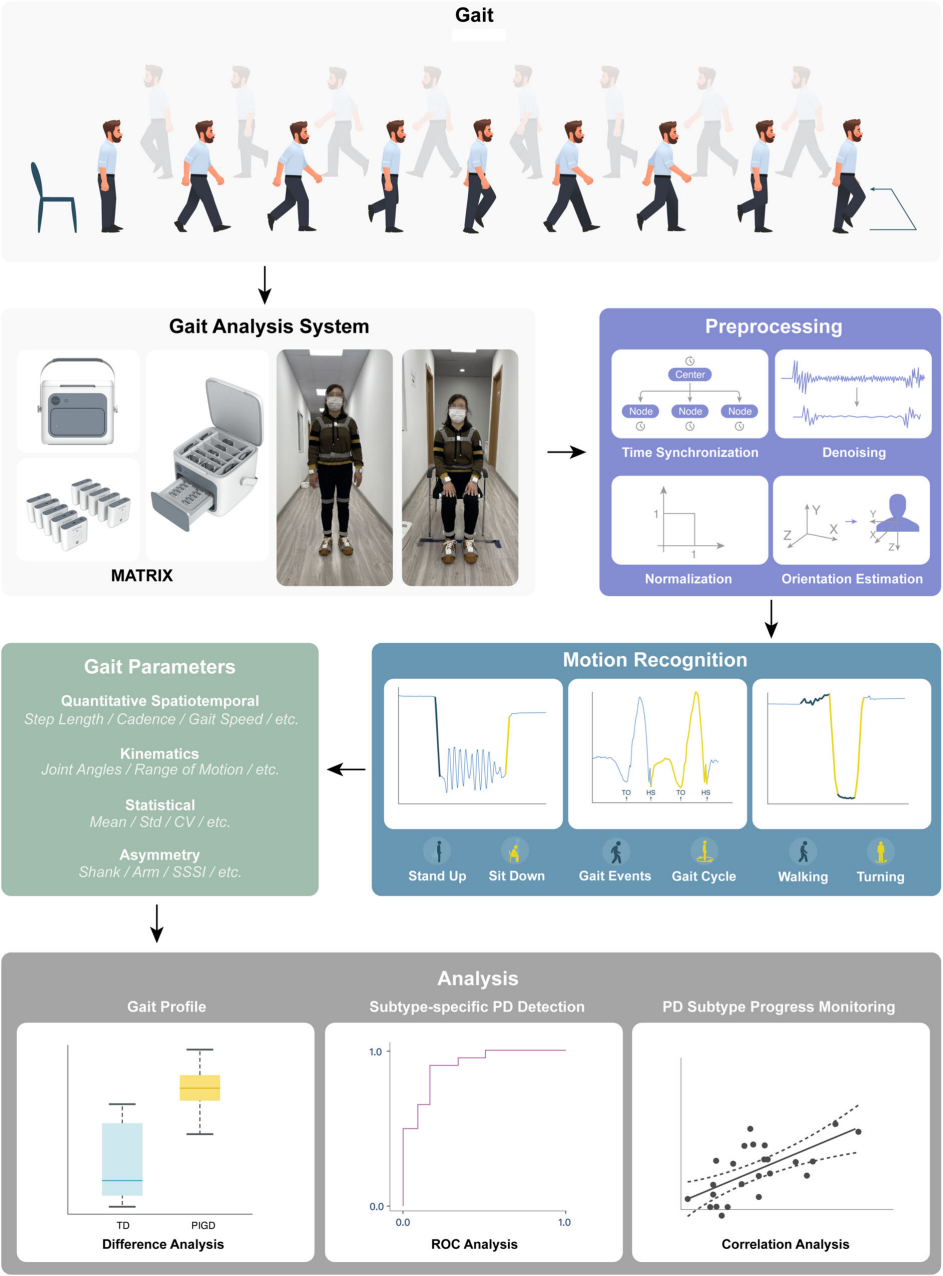
Graphical synopsis of wearable sensor-based gait analysis in Parkinson’s disease subtypes. The diagram shows the entire process of gait parameters acquisition, data processing, and data analysis. Difference analysis, ROC analysis, and correlation analysis are used to explore the objective gait alterations in early PD subtypes and to identify the potential gait biomarkers for early diagnosis, subtype differentiation, and disease severity monitoring. Here, the box plot is used to represent difference analysis (the center line and box indicate the median and interquartile range, respectively; the whiskers extend to the minimum and maximum). TD tremor-dominant, PIGD postural instability and gait disorder, TO Toe Off, HS Heel Strike, ROC receiver operating characteristic.

Therefore, in this study, we extended our analysis to early PD patients with TD and PIGD subtype and applied ten body-fixed sensors (Fig. 1) during the TUG test, aiming to characterize comprehensive (upper/lower limbs, trunk, and lumbar, postural transitions) and objective gait alterations in early PD subtypes. Moreover, the potential gait markers for early diagnosis and subtype differentiation were investigated. The correlation analysis between gait features and disease severity was further conducted in PD subtypes for identifying potential subtype-specific gait markers for disease severity monitoring. The study synopsis is shown in Fig. 2.

## Results

### Demographic characteristics and clinical evaluation

The demographic information and clinical characteristics are described in Table 1. The age, gender, and figures, including height, weight, and BMI, were matched among TD, PIGD, and HC groups. PD patients with both TD and PIGD subtypes had slightly lower Mini-mental State Examination (MMSE) scores when compared with the educational levels matched HC, there were no significant but trends of statistical difference in *p* values after Bonferroni correction in multiple comparisons (*p* = 0.07 and 0.081). Disease duration and severity measured by MDS-UPDRS and Hoehn-Yahr (H-Y) stage between TD and PIGD patients were both matched. In addition, patients with TD and PIGD showed no significant difference in Berg Balance Scale (BBS) score at the early stage.

**Table 1:** Clinical data of the HC and of the TD and PIGD patients

Characteristic	HC (*n* = 39)	TD (*n* = 24)	PIGD (*n* = 20)	*p* value
Age, year	66 (56~68)	61.63 ± 7.17	62.05 ± 9.61	0.663^a^
Gender, M/F	22/17	15/9	11/9	0.855^a^
Height, cm	163.77 ± 7.84	166.83 ± 7.22	166 ± 8.86	0.292^a^
Weight, kg	66 (56~75)	67.65 ± 7.9	68.23 ± 13.47	0.789^a^
BMI (kg/m^2^)	25.2 ± 3.53	24.23 ± 1.57	24.54 ± 2.86	0.62^a^
Education year	9 (9~12)	12 (9.75~13)	12 (9~12.75)	0.202^a^
MMSE	28 (27~29)	27.21 ± 1.44	26.68 ± 2.51	**0.025**^a^
Duration, months	-	24 (18.25~60)	21 (12~36)	0.139^b^
BBS	-	55 (54~56)	55 (52~56)	0.912^b^
MDS-UPDRS total score	-	39.63 ± 20.99	31.75 ± 16.28	0.178^b^
Part I score	-	5.46 ± 4.27	4.9 ± 3.23	0.633^b^
Part II score	-	8.46 ± 5.31	8.05 ± 4.1	0.78^b^
Part III score	-	25.71 ± 14.87	17.5 (11.25~24.25)	0.075^b^
H-Y stage				0.685^b^
1		11	7	
1.5		4	2	
2		6	8	
2.5		3	3	

*HC* healthy controls, *TD* tremor-dominant, *PIGD* postural instability and gait disorder, *BMI* body mass index, *MMSE* Mini-mental State Examination, *BBS* Berg Balance scale, *MDS-UPDRS* Movement Disorders Society-Unified Parkinson’s Disease Rating Scale, *H-Y stage* Hoehn and Yahr stage. Data are displayed as mean ± standard deviation or median (quartile[Q]1~Q3). ^a^*p* value for the comparison among the HC, TD, and PIGD groups. ^b^*p* value for the comparison between the TD and PIGD groups. *p* values for the pair-wise comparison after the Bonferroni correction were 0.07 (HC vs. TD), 0.081(HC vs. PIGD), and 1(TD vs. PIGD) in the multiple comparisons of the MMSE. Bold values are marked as significant.

### Potential gait biomarkers for early diagnosis of PD subtypes

Early and subtle gait alterations were detected in both PD subtypes, which could be potential objective gait biomarkers for early diagnosis (Table 2). The gait parameters with high diagnostic value in differentiating PD subtypes from the HC were identified by the receiver operating characteristic (ROC) analysis (Supplementary Table 1). These gait features include: (1) Lower limbs: PD patients with both subtypes showed more swing and less stance time in the more affected side (MAS) lower limb. The ROC analysis revealed the area under the curve (AUC) values more than 0.7 (*p* < 0.05) for the MAS Swing and MAS Stance. In addition, both PD subtypes showed a smaller backswing range in bilateral lower extremities than the HC did. Therefore, MAS and less affected side (LAS) Shank Backward Swing Maximum also presented high diagnostic value with the AUC of 0.779–0.845 (*p* ≤ 0.001) in PD subtypes. The smaller peak angular velocity of the MAS lower limb and shank symbolic symmetry index were found in both TD and PIGD groups than that in the control group, although only the differences between TD patients and the controls still showed significance after the correction in multiple comparison (*p* = 0.043 and 0.014), and no difference was found in the comparison between the PD subtypes. (2) Trunk and lumbar: the range of motion in the coronal plane of the trunk and lumbar were smaller in both PD subtypes than that in the HC, with the AUC of 0.716–0.864 (*p* < 0.01) for Trunk Coronal RoM and 0.796–0.871 (*p* < 0.001) for Lumbar Coronal RoM. (3) Upper limbs: compared with the HC, the backswing range of the MAS arm was significantly reduced in both PD subgroups. The ROC analysis revealed AUC values of 0.871 and 0.879 (*p* < 0.001) for the MAS Arm Backward Swing Maximum, which showed the highest diagnostic value in both PD subtypes. Moreover, the Arm Symbolic Symmetry Index presented greater in early PD patients than that in the HC, with AUC values of 0.762–0.868 (*p* ≤ 0.001). (4) Postural transitions: During the turning process, both PD subtypes presented longer duration and slower average angular velocity than the HC (*p* < 0.05), with the AUC values more than 0.7 (*p* < 0.05) for these features in both PD subtypes revealed by the ROC analysis. Patients with both PD subtypes completed the positional transition with a slower trunk sagittal peak velocity compared with the HC when sitting down and standing up, with the AUC values of 0.709–0.816 (*p* < 0.01) for the corresponding features in the ROC analysis. In addition, the maximum of the range of trunk lean backward when sitting down in both PD subtypes were smaller than that in the HC and the ROC analysis showed high AUC values of 0.744–0.789 (*p* < 0.01) for this feature in differentiating PD subtypes from the HC.

**Table 2:** Gait characteristics of the HC and of the TD and PIGD patients

Gait parameters	HC (*n* = 39)	TD (*n* = 24)	PIGD (*n* = 20)	*p* value			
				HC vs. TD vs. PIGD	HC vs. TD	HC vs. PIGD	TD vs. PIGD
Lower body-related parameters							
MAS Step Length (cm)	58.26 (56.07~63.94)	59.27 (53.83~62.8)	51.7 (48.83~67.12)	0.137			
LAS Step Length (cm)	58.26 (56.07~63.94)	59.26 (53.81~63.22)	54.68 ± 9.86	0.116			
MAS Stride Velocity (m/s)	1.07 ± 0.17	1.03 ± 0.14	0.99 ± 0.22	0.288			
LAS Stride Velocity (m/s)	1.07 ± 0.17	1.03 ± 0.14	0.99 ± 0.2	0.257			
MAS Stride Length (cm)	114.87 (110.6~126.51)	115.62 (104.37~122.76)	108.49 ± 20	0.181			
LAS Stride Length (cm)	114.87 (110.6~126.51)	112.86 ± 13.66	108.19 ± 19.19	0.139			
MAS Gait Cycle time (s)	1.11 ± 0.08	1.11 ± 0.08	1.12 ± 0.1	0.649			
LAS Gait Cycle time (s)	1.11 ± 0.08	1.11 ± 0.08	1.12 ± 0.09	0.687			
MAS Cadence (step/min)	109.64 ± 7.9	106.7 ± 9.29	107.19 (101.69~110.54)	0.204			
LAS Cadence (step/min)	109.64 ± 7.9	113.27 ± 8.57	110.13 ± 10.88	0.127			
MAS Double Support (%)	22.24 ± 3.18	20.95 ± 4.23	20.56 ± 5.04	0.198			
LAS Double Support (%)	22.24 ± 3.18	21.46 ± 3.63	20.72 ± 4.37	0.288			
MAS Swing (%)	39.38 ± 1.61	41.28 ± 2.56	40.97 ± 2.29	**0.003**	**0.007**	**0.044**	1
LAS Swing (%)	39.38 ± 1.61	38.83 ± 2.6	39.56 ± 2.92	0.512			
MAS Stance (%)	60.62 ± 1.61	58.72 ± 2.56	59.03 ± 2.29	**0.003**	**0.007**	**0.044**	1
LAS Stance (%)	60.62 ± 1.61	61.17 ± 2.6	60.44 ± 2.92	0.512			
MAS Shank Forward Swing Maximum (°)	23.31 ± 5.86	20.68 ± 6.58	21.67 ± 8.9	0.266			
LAS Shank Forward Swing Maximum (°)	23.31 ± 5.86	22.69 ± 6.48	23.02 ± 7.39	0.924			
MAS Shank Backward Swing Maximum (°)	−47.42 (−50.22~−45.72)	−42.05 ± 4.33	−42.17 ± 5.27	**<0.001**	**<0.001**	**0.002**	1
LAS Shank Backward Swing Maximum (°)	−47.42 (−50.22~−45.72)	−41.56 ± 4.59	−41.4 ± 5.05	**<0.001**	**<0.001**	**<0.001**	1
MAS Peak Shank Angular Velocity(°/s)	329.07 ± 44.48	302.58 ± 33.62	307.55 ± 60.65	**0.033**	**0.043**	0.265	1
LAS Peak Shank Angular Velocity (°/s)	329.07 ± 44.48	324.01 ± 41.7	317.04 ± 51.21	0.671			
Stride Velocity Asymmetry (%)	6.9 ± 2.76	6.09 ± 2.65	6.35 ± 2.21	0.546			
Stride Length Asymmetry (%)	4.23 (3.51~6.7)	4.83 ± 2.18	4.24 ± 1.56	0.441			
Swing Asymmetry (%)	8.18 ± 2.47	9.24 (6.78~12.54)	7.49 ± 3.09	0.136			
Stance Asymmetry (%)	5.39 ± 1.76	6.07 (4.27~8.33)	5.06 ± 2	0.101			
Shank RoM Asymmetry (%)	9.39 (4.47~12.82)	14.33 ± 8.79	10.57 ± 6.05	0.077			
Peak Shank Angular Velocity Asymmetry (%)	9.97 (6.2~14.52)	14.55 ± 8.63	10.81 ± 5.73	0.355			
Shank Symbolic Symmetry Index (%)	11.6 (10.51~12.77)	10.09 (8.25~11.97)	10.73 ± 2.11	**0.013**	**0.014**	0.216	1
Mean Phase Difference (%)	3.29 (2.44~3.94)	3.9 (2.32~6.56)	3.79 ± 1.62	0.404			
Phase Coordination Index (%)	6 (4.77~8.74)	6.76 (4.59~10.26)	6.83 ± 2.26	0.755			
Trunk and lumbar related parameters							
Trunk Coronal Peak Velocity (°/s)	25.74 (20.33~28.65)	23.55 ± 5.59	19.19 (16.58~23.71)	**0.016**	1	**0.013**	0.185
Trunk Coronal RoM (°)	5.7 ± 1.92	4.3 ± 1.73	3.41 ± 1.15	**<0.001**	**0.013**	**<0.001**	0.262
Trunk Sagittal Peak Velocity (°/s)	36.43 ± 7.33	31.74 (27.55~36.95)	28.49 (25.99~37.07)	**0.005**	0.097	**0.007**	1
Trunk Sagittal RoM (°)	5.31 ± 1.05	5.03 ± 1.04	4.47 (3.69~5.23)	0.053			
Trunk Transverse Peak Velocity (°/s)	44.03 ± 9.71	38.23 ± 6.82	37.04 ± 11.34	**0.009**	0.075	**0.017**	1
Trunk Transverse RoM (°)	11.06 ± 2.69	10.21 ± 2.75	9.67 ± 2	0.179			
Lumbar Coronal Peak Velocity (°/s)	44.7 ± 15.03	36.08 ± 14.65	32.32 (24.22~51.18)	**0.036**	0.108	0.094	1
Lumbar Coronal RoM (°)	5.75 ± 1.51	3.8 ± 0.97	4 ± 1.71	**<0.001**	**<0.001**	**<0.001**	1
Lumbar Sagittal Peak Velocity (°/s)	69.51 (48.1~136.38)	59.08 (44.58~71.37)	47.85 (32.9~88.53)	**0.033**	0.373	**0.034**	0.98
Lumbar Sagittal RoM (°)	7.32 (5.06~9.54)	5.64 (4.56~6.62)	5.26 (4.43~6.42)	0.058			
Lumbar Transverse Peak Velocity (°/s)	56.21 (50.85~81)	56.73 ± 14.16	51.61 ± 15.9	0.059			
Lumbar Transverse RoM (°)	9.13 ± 2.25	10.14 ± 2.62	8.61 (7.28~11.39)	0.334			
Upper body related parameters							
MAS Arm Peak Velocity (°/s)	175.97 ± 63.18	155.14 (107.64~241.7)	100.04 (73.97~118.48)	**<0.001**	1	**<0.001**	**0.002**
LAS Arm Peak Velocity (°/s)	175.97 ± 63.18	177.6 ± 47.52	117.2 (84.33~163.8)	**0.009**	1	**0.02**	**0.016**
MAS Arm Forward Swing Maximum (°)	38.24 (28.07~49.77)	33.04 (28.85~36.39)	32.62 ± 10.31	0.214			
LAS Arm Forward Swing Maximum (°)	38.24 (28.07~49.77)	40.21 ± 12.37	35.03 ± 10.84	0.209			
MAS Arm Backward Swing Maximum (°)	0.12 (−12.29~5.5)	15.92 ± 10.54	14 ± 9.41	**<0.001**	**<0.001**	**<0.001**	1
LAS Arm Backward Swing Maximum (°)	0.12 (−12.29~5.5)	1.58 ± 6.84	7.23 ± 8.93	**0.009**	1	**0.007**	0.098
Arm Velocity Asymmetry (%)	20.03 (14.18~26.81)	35.37 ± 17.34	20.57 (16.72~30.37)	**0.009**	**0.008**	1	0.127
Arm Symbolic Symmetry Index (%)	33.74 ± 3.96	41.83 ± 6.52	38.07 (34.59~40.35)	**<0.001**	**<0.001**	**0.044**	0.131
Postural transitions-related parameters							
Turning-Average Duration (s)	1.43 (1.41~1.59)	1.49 (1.44~1.76)	1.54 (1.46~2.1)	**<0.001**	**0.02**	**0.001**	0.892
Turning-Peak Velocity (°/s)	157.52 ± 23.63	142.49 ± 24.62	125.06 ± 30.89	**<0.001**	0.133	**<0.001**	0.175
Turning-Average Step Duration (s)	0.53 ± 0.07	0.51 ± 0.06	0.56 ± 0.05	**0.041**	1	0.199	**0.038**
Turning-Average Angular Velocity(°/s)	126.12 (116.13~128.09)	121.54 (106~125.32)	117.82 (90.69~123.78)	**<0.001**	**0.018**	**<0.001**	0.84
Turning-Average Steps	2.09 ± 0.41	2.5 (2.19~3)	2.42 (1.91~2.75)	**0.003**	**0.004**	0.076	1
SiSt - Duration (s)	1.73 (1.34~2.14)	1.52 (1.32~1.78)	1.56 (1.24~1.95)	0.295			
SiSt-Trunk Sagittal Peak Velocity(°/s)	85.06 (72.76~105.85)	66.22 ± 12.75	59.69 (51.43~83.71)	**<0.001**	**<0.001**	**<0.001**	1
SiSt-Trunk Lean Backward Maximum (°)	20.85 ± 8.88	14.5 ± 9.41	16.2 ± 9.88	**0.021**	**0.027**	0.204	1
SiSt-Trunk Lean Forward Maximum (°)	−19.45 ± 11.83	−20.56 ± 12.18	−18.4 ± 12.94	0.733			
StSi - Duration (s)	2.38 (1.91~2.82)	1.74 (1.56~1.87)	2.06 ± 0.53	**<0.001**	**<0.001**	0.185	0.297
StSi-Trunk Sagittal Peak Velocity (°/s)	79.16 ± 22.36	61.53 ± 18.9	60.69 (51.9~66.59)	**0.003**	**0.015**	**0.014**	1
StSi- Trunk Lean Backward Maximum (°)	20.8 ± 10.01	10.14 ± 9.87	12.54 ± 9.3	**<0.001**	**<0.001**	**0.008**	1
StSi-trunk lean forward maximum (°)	−18.86 ± 14.44	−21.89 ± 13.71	−19.84 ± 11.69	0.673			

*HC* healthy controls, *TD* tremor-dominant, *PIGD* postural instability and gait disorder, *MAS* more affected side, *LAS* less affected side, *RoM* range of motion, *SiSt* sit to stand, *StSi* stand to sit. Data are displayed as Mean ± Standard Deviation or Median (quartile[Q]1~Q3). Kruskal-Wallis test was used to compare various gait parameters among the HC, TD and PIGD groups. Pair-wise comparisons were corrected for multiple comparisons using Bonferroni method. Bold values are marked as significant.

**Fig. 3:**
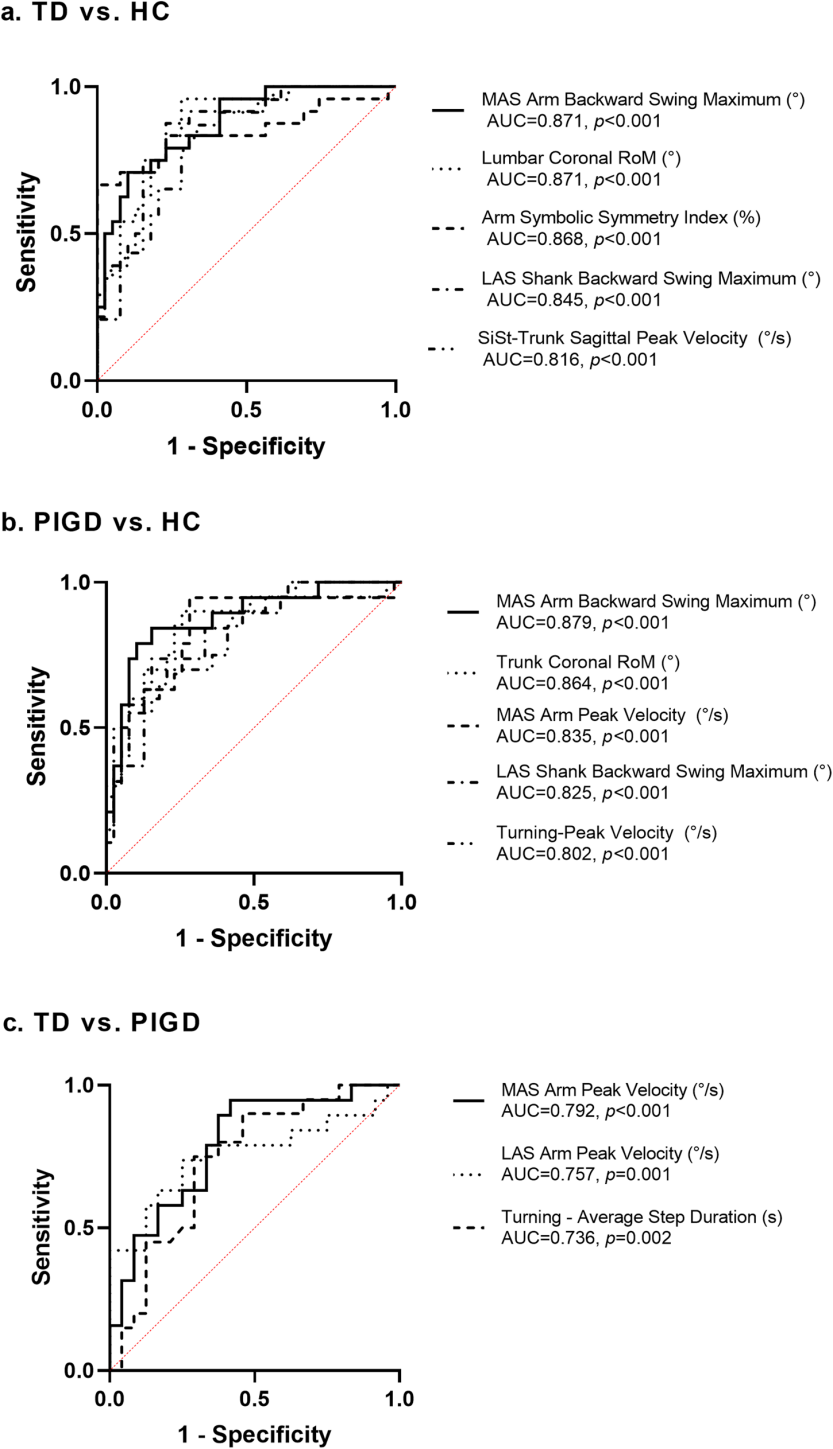
The receiver operating characteristics (ROC) curves for the gait metrics with the best discriminative value between Parkinson’s Disease (PD) subtypes and healthy controls (HC), as well as between tremor-dominant (TD) and postural instability and gait disorder (PIGD) subtype. The ROC curves for the gait metrics with the best discriminative value between **a** TD and HC; **b** PIGD and HC; **c** TD and PIGD. AUC value is reported for each curve. MAS more affected side, LAS less affected side, RoM range of motion, SiSt sit to stand. Note, if the same gait parameter showed significantly discriminative ability in both MAS and LAS limbs, only the side with higher AUC value was selected.

Apart from the same gait alterations in PD subtypes, several subtype-specific gait features were identified which could be helpful for early subtype diagnosis (Table 2, Supplementary Table 1). (1) Trunk and lumbar: patients with PIGD subtype presented decreased velocity of the trunk and lumbar motion in most directions with the AUC values more than 0.7 (*p* < 0.05) for these features revealed by the ROC analysis. (2) Upper limbs: The peak velocities of bilateral arms were slower in PIGD patients than those in HC and TD groups, and the ROC analysis showed high AUC values of 0.715–0.835 (*p* < 0.01) for the MAS/LAS Arm Peak Velocity in differentiating PIGD from the HC. The arm velocity asymmetry was higher in the TD group than that in the HC, and the ROC analysis showed AUC values of 0.716 (*p* < 0.01) for this feature in differentiating TD from the HC. (3) Postural transitions: Turning-Average Steps and SiSt-Trunk Lean Backward Maximum of TD subtype and the Turning-Peak Velocity of PIGD subtype were significantly discriminative in differentiating TD and PIGD subtype from the HC with the AUC values of more than 0.7 (*p* < 0.01). The ROC curves of the AUC > 0.8 for the gait parameters in differentiating TD and PIGD patients from the HC were shown in Fig. 3a, b, respectively.

### Early differentiation between TD and PIGD subtypes using objective gait features

No significant difference in clinical scales (such as MDS-UPDRS and H-Y scale) and clinical gait tests (such as BBS) between early TD and PIGD patients, however, three gait features resulted in statistically significant differences between these two PD motor subtypes (Table 2, Supplementary Table 1, Fig. 3c) : (1) Upper limbs: when compared with the TD group, the PIGD group showed slower bilateral arm peak velocity, and the MAS Arm Peak Velocity could best distinguish TD from PIGD with the AUC of 0.792 (*p* < 0.001), followed by LAS Arm Peak Velocity with the AUC of 0.757 (*p* = 0.001). (2) Postural transitions: during the turning process, the average step duration of patients with PIGD subtype was longer than that of patients with TD subtype. The ROC analysis showed the AUC of 0.736 (*p* = 0.002) for Turning-Average Step Duration in subtype differentiation. Clearly, the gait damage in the PIGD group is more serious than that in the TD group based on the above results of subgroup comparison (Table 2). Moreover, subtle but measurable gait differences existed between TD and PIGD patients before the onset of clinically apparent gait impairment, which could be the potential diagnostic markers for early subtype distinction.

### Disease severity monitoring for PD subtypes based on objective gait biomarkers

**Fig. 4:**
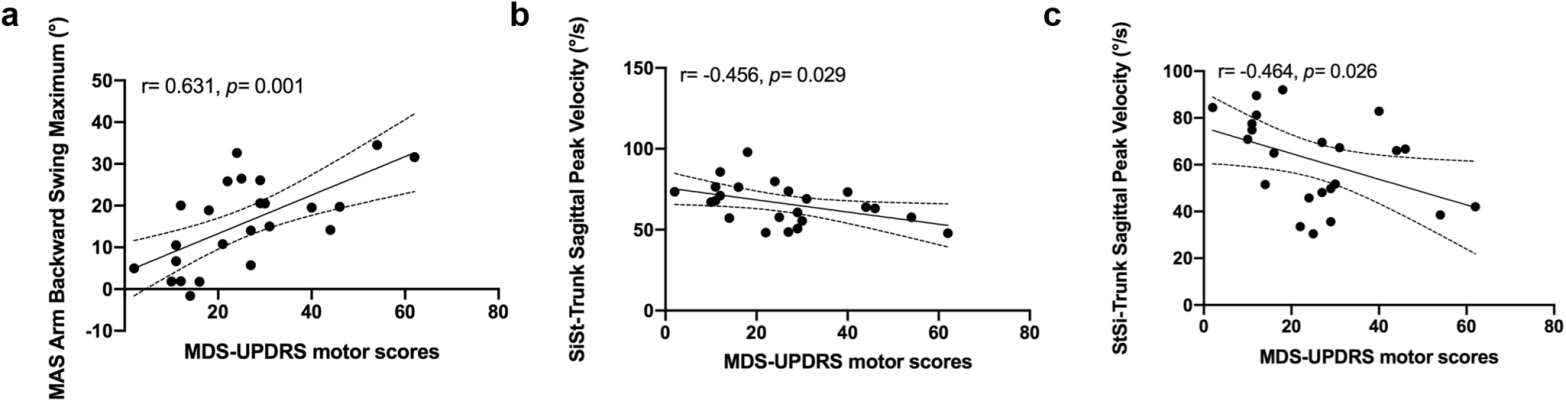
The relationship between Movement Disorders Society-Unified Parkinson’s Disease Rating Scale (MDS-UPDRS) motor scores and specific gait parameters in the tremor-dominant (TD) group. Correlation between MDS-UPDRS motor scores and **a** MAS Arm Backward Swing Maximum, **b** SiSt-Trunk Sagittal Peak Velocity, **c** StSi-Trunk Sagittal Peak Velocity. MAS more affected side, SiSt sit to stand, StSi stand to sit.

**Fig. 5:**
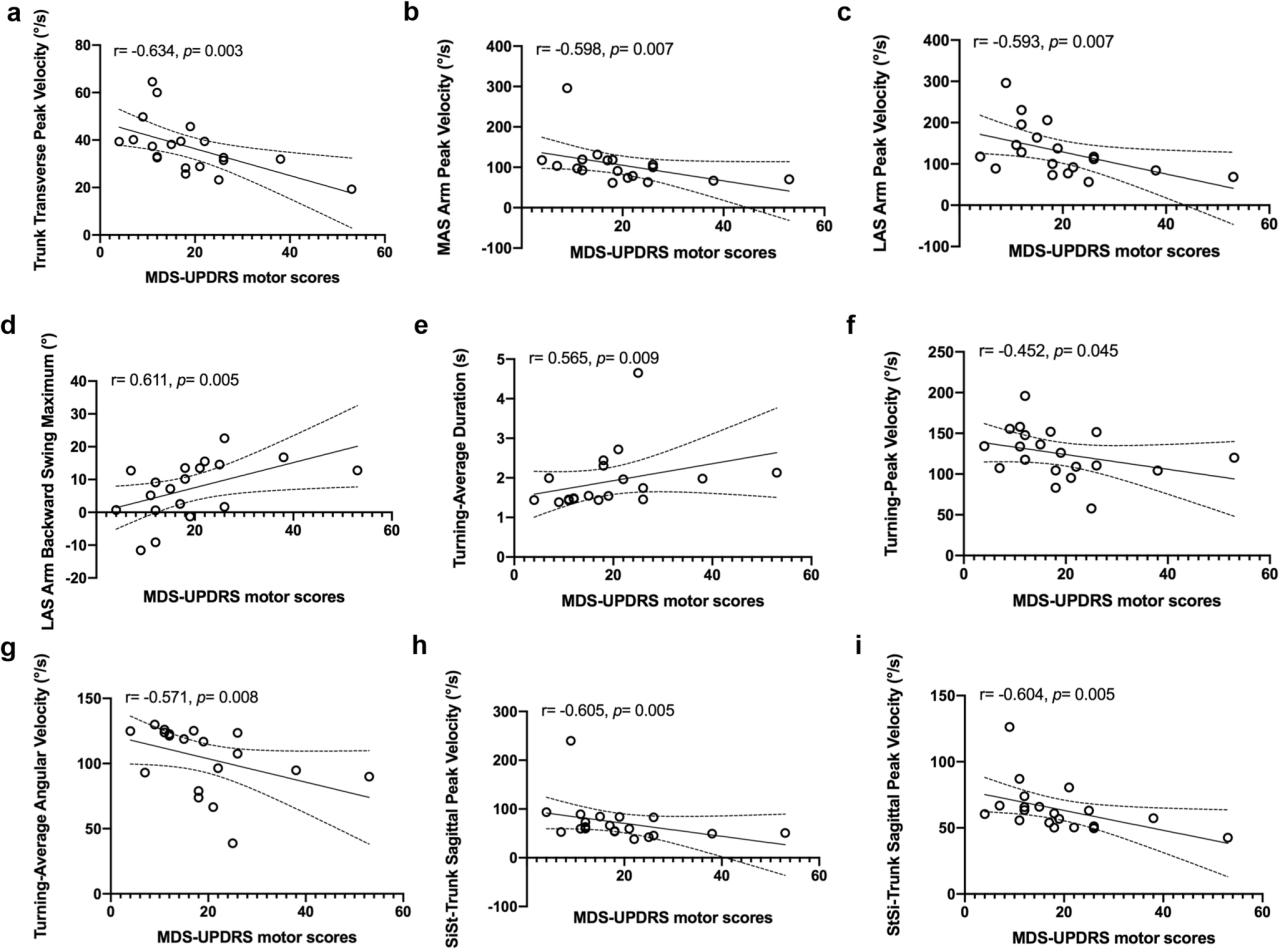
The relationship between Movement Disorders Society-Unified Parkinson’s Disease Rating Scale (MDS-UPDRS) motor scores and specific gait parameters in the postural instability and gait disorder (PIGD) group. Correlation between MDS-UPDRS motor scores and **a** Trunk transverse peak velocity, **b** MAS arm peak velocity, **c** LAS arm peak velocity, **d** LAS arm backward swing maximum, **e** Turning-average duration, **f** Turning-peak velocity, **g** Turning-average angular velocity, **h** SiSt-trunk sagittal peak velocity and **i** StSi-trunk sagittal peak velocity. MAS more affected side, LAS less affected side, SiSt sit to stand, StSi stand to sit.

The correlations between the disease severity measured by the MDS-UPDRS motor scores and gait parameters for PD subtypes were showed in Table 3 and Figs. 4, 5. (1) Trunk and lumbar: Trunk Transverse Peak Velocity was significantly correlated with MDS-UPDRS motor scores in PIGD subtype (*r* = −0.634, *p* = 0.003). (2) Upper limbs: MAS Arm Backward Swing Maximum was significantly correlated with MDS-UPDRS motor scores in TD subtype (*r* = 0.631, *p* = 0.001). While, bilateral Arm Peak Velocity (*r* = −0.598, *p* = 0.007; *r* = −0.593, *p* = 0.007) and LAS Arm Backward Swing Maximum (*r* = 0.611, *p* = 0.005) were significantly correlated with disease severity in PIGD subtype. (3) Postural transitions: SiSt/StSi-Trunk Sagittal Peak Velocity showed moderate correlations (*p* < 0.05) with the disease severity in both PD subtypes. In addition, for PIGD patients, the Duration (*r* = 0.565, *p* = 0.009), Peak Velocity (*r* = −0.452, *p* = 0.045), and average angular velocity (*r* = −0.571, *p* = 0.008) during the turning process were also significantly correlated with disease severity.

**Table 3:** Correlation between selected gait variables^a^ and MDS-UPDRS motor scores of patients with PD subtypes

Gait parameters	TD		PIGD	
	*r*	*p*	*r*	*p*
Lower body-related gait parameters				
MAS Swing (%)	0.273	0.196	0.239	0.324
MAS Stance (%)	−0.273	0.196	−0.239	0.324
MAS Shank Backward Swing Maximum (°)	0.011	0.96	−0.164	0.503
LAS Shank Backward Swing Maximum (°)	−0.282	0.182	0.055	0.822
MAS Peak Shank Angular Velocity(°/s)	−0.392	0.058		
Shank Symbolic Symmetry Index (%)	−0.166	0.438		
Trunk and lumbar−related gait parameters				
Trunk Coronal Peak Velocity (°/s)			−0.152	0.524
Trunk Coronal RoM (°)	−0.138	0.519	−0.277	0.236
Trunk Sagittal Peak Velocity (°/s)			−0.351	0.13
Trunk Transverse Peak Velocity (°/s)			**−0.634**	**0.003**
Lumbar Coronal RoM (°)	−0.147	0.494	−0.076	0.75
Lumbar Sagittal Peak Velocity (°/s)			−0.424	0.063
Upper body-related gait parameters				
MAS Arm Peak Velocity (°/s)			**−0.598**	**0.007**
LAS Arm Peak Velocity (°/s)			**−0.593**	**0.007**
MAS Arm Backward Swing Maximum (°)	**0.631**	**0.001**	0.347	0.145
LAS Arm Backward Swing Maximum (°)			**0.611**	**0.005**
Arm Velocity Asymmetry (%)	0.187	0.381		
Arm Symbolic Symmetry Index (%)	0.373	0.073	0.03	0.9
Postural transitions related gait parameters				
Turning-Average Duration (s)	0.382	0.065	**0.565**	**0.009**
Turning-Peak Velocity (°/s)			**−0.452**	**0.045**
Turning-Average Step Duration (s)				
Turning-Average Angular Velocity (°/s)	−0.376	0.07	**−0.571**	**0.008**
Turning-Average Steps	0.27	0.202		
SiSt-Trunk Sagittal Peak Velocity (°/s)	**−0.456**	**0.029**	**−0.605**	**0.005**
SiSt-Trunk Lean Backward Maximum (°)	−0.27	0.214		
StSi- Duration (s)	0.079	0.721		
StSi-Trunk Sagittal Peak Velocity (°/s)	**−0.464**	**0.026**	**−0.604**	**0.005**
StSi-Trunk Lean Backward Maximum (°)	−0.295	0.172	−0.079	0.74

*PD* Parkinson’s disease, *MDS-UPDRS* Movement Disorders Society-Unified Parkinson’s Disease Rating Scale, *TD* tremor-dominant, *PIGD* postural instability and gait disorder, *MAS* more affected side, *LAS* less affected side, *RoM* range of motion, *SiSt* sit to stand, *StSi* stand to sit. Spearman’s correlation coefficient (*r*) calculated for TD and PIGD patients. ^a^gait variables with significant Kruskal Wallis test and the corresponding pair-wise comparison. Bold values are marked as significant.

**Fig. 6:**
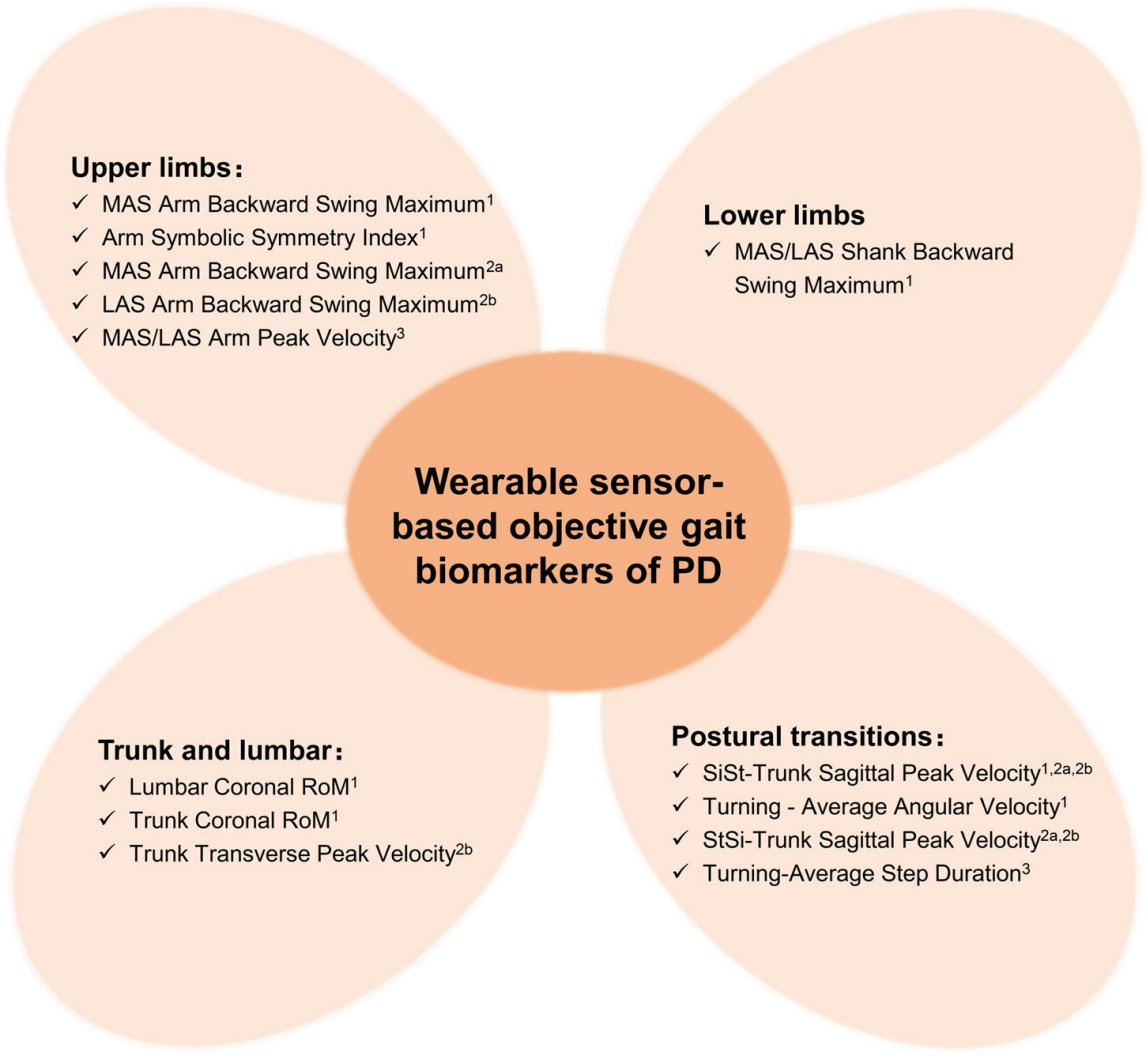
Wearable sensor-based objective gait biomarkers of Parkinson’s disease (PD). The recommended objective gait features for early diagnosis, subtype differentiation, and disease monitoring are listed in this figure. TD tremor-dominant, PIGD postural instability and gait disorder, MAS more affected side, LAS less affected side, RoM range of motion, SiSt sit to stand, StSi stand to sit.^1^ Gait features for early detection of both TD and PIGD (for subtype-specific early detection details, please see Fig. 3 and Supplementary Table 1);^2a^ Gait features for disease monitoring in TD subtype;^2b^ Gait features for disease monitoring in PIGD subtype; ^3^Gait features for subtypes differentiation.

Based on the results above, we further summarized the recommended objective gait biomarkers for early diagnosis, subtype differentiation, and disease monitoring. The gait biomarkers related to upper/lower limbs, trunk and lumbar or postural transitions were listed, respectively, in Fig. 6.

## Discussion

The present study provides a relatively comprehensive description of early gait alterations in the TD and PIGD subtypes of PD. Our key findings were: (1) Wearable sensor-based objective gait metrics may be the potential gait biomarkers for early diagnosis of PD or its specific subtype. (2) The gait damage in the PIGD group is more serious than that in the TD group, and bilateral arm peak velocity could best distinguish between TD and PIGD subtypes. (3) Specific gait metrics correlate with disease severity in PD subtypes, which may aid in quantitative disease severity monitoring.

Our study proved that wearable sensor-based gait analysis could detect subtle but measurable gait alterations in early PD subtypes. These features may be the potential gait biomarkers for early diagnosis of PD or its specific subtype. (1) Lower limbs: In our study, we found that the backswing range of the lower limbs was significantly reduced in both PD subtypes. Particularly, the LAS shank showed a higher discriminative value for early diagnosis. Our finding was congruent with the notion that the alterations in the least affected leg suggested that PD patients attempted to enhance global dynamic balance and attenuate the increased trunk rotation as compensation for absent arm swing^46^. In addition, interestingly, the reduced proportion of swing to stance was reported in PD patients in a previous study by Knutsson^47^, which is in congruent with the traditional notion that PD patients are supposed to have decrease in the swing phase because of the rigidity and increase in stance time to keep balance while walking. However, in the present study, the MAS lower body showed a slightly increased swing and corresponding decreased stance in both PD subtypes. Notably, most of the PD patients were in an advanced stage in Knutsson’s study^47^, and also, the difference of the bilateral limbs was not fully studied. Combined with the above, we considered our finding perhaps presented a compensatory gait in early PD in which the rigidity was not enough to disrupt the biomechanics of PD patients. Therefore, the MAS lower limb tend to augment the swing to maintain the coordination with the LAS limb. Further studies with a follow-up cohort are needed to confirm this finding. Galna et al. found that the stride velocity of PD patients was slower than that of the controls, and the change was subtle over 18 months^9^. However, in our study, the stride velocities of both PD subgroups were not significantly decreased compared with the control group. The disease severity may account for the different results. In the study by Galna et al.^9^, the patients were in the H-Y stages of 1–3, and 9% of PD patients presented FOG. Conversely, in our study, most PD patients were in the H-Y stage of 1–2, and patients with FOG were not included in this study. And our finding was consistent with the previous study by Zampieri et al.^26^ in which the mean H-Y stage of PD subjects was 1.6 (range: 1–2.5) and the stride velocity showed no significant difference between PD and HC. Therefore, changes in gait speed, which may also be observed in other disorders^48,49^ as well as in older adults^50^, is not sensitive and specific in differentiating PD patients at the early stage. Similarly, other common gait alterations in PD patients, such as gait cycle time and step length, showed no significant difference between PD subtypes and the HC. Although the result seemed to be inconsistent with our clinical observation, we could speculate the conventional gait features may not differentiate PD from the HC in early stage of the disease. (2) Trunk and lumbar: The mobility of trunk and lumbar was limited in both velocity, and the range of motion in PD, and such variables were with high discriminative values. In congruent with the findings by Zampieri et al.^26^, who also reported a high AUC for peak trunk rotation velocity (0.806) and trunk rotation range of motion (0.764) in differentiating PD from the HC. Moreover, in our study, we used two sensors to detect the mobility of the trunk and lumbar, respectively. As a result, more sensitive and comprehensive findings were found in different motor subtypes of PD. The most sensitive trunk and lumbar-related gait parameters in TD and PIGD subtypes were the coronal range of motion of the lumbar and the trunk, respectively. A significant decrease in peak velocity of the trunk and lumbar rotation was accompanied by in PIGD patients, indicating that the PIGD patients had widespread trunk impairments and worse trunk flexibility. In a previous article^51^, the researchers concluded that the spinal configuration and flexibility might be altered in early PD before frank postural instability occurred. Schenkman and co-workers also implicated that spinal flexibility is an established measure of balance control in early PD patients^52^. Therefore, we could infer reasonably that impaired spinal flexibility and balance control lead to evident postural instability and gait impairment in the PIGD group. (3) Upper limbs: The backswing range of the upper limbs was significantly reduced in both PD subtypes, and the most sensitive gait deficit in the early stage of PD was the restricted retroversion of the MAS arm. Our results were supported by Roggendorf’s study, in which the authors analyzed the decomposed arm swing and implicated the impaired active arm retroversion as the earliest sign of upper extremity dysfunction in Parkinsonian gait^53^. However, the forward swing range was not significantly affected, and we speculate that the decrease in the backswing range may be due to the increased flexor tension of bulky muscle in the proximal limbs, which resulted in the insufficiency of limb extension. Additionally, in line with the previous work^54,55^, the arm symbolic symmetry index that refers to the asymmetry of arm swing amplitude also showed a discriminative value in differentiating both TD and PIGD groups from the control group because of the asymmetry of motor symptoms in PD patients. Moreover, the TD group presented a relatively more obvious asymmetry in arm swing. It has been proven that arm swing is particularly diminished on the more affected body side, and the arm swing asymmetry attenuates with disease progression^53^. Therefore, bilateral arm swing has been impaired in the PIGD group even in a relatively early disease stage. The arm peak velocity was not significantly different in the TD group compared to the control group, although the decreased trend in the MAS arm led to a greater arm velocity asymmetry which was also observed in the previous study^23^. For PIGD patients, MAS arm peak velocity was significantly reduced and also with high discriminative value. These results are partially in line with the findings by Zampieri et al.^26^, although the gait characteristics of different motor subtypes have not been analyzed separately in their study. (4) Postural transitions: During the turning process, the average turning duration and average angular velocity showed high discriminative values for distinguishing the TD and PIGD groups from the control group, which indicated the low turning efficiency in both PD subtypes. Low turning efficiency was also reported in other studies^26,56^, in which they showed high AUC values for turning duration (0.89) and average turning velocity (0.764) in discriminating the PD patients from the HC, respectively. In addition, we found peak turning velocity was the most sensitive variable of turning related gait parameter to differentiate PIGD patients from the HC, however, no significant difference in peak turning velocity between TD patients and the HC was found. Our results suggest the spectrum of gait abnormalities varies in early-stage PD patients with different motor subtypes. During the processes of standing up and sitting down, the peak velocity of the trunk in sagittal plane significantly reduced in both TD and PIGD patients, which is consistent with the findings of a previous study^26^, and both variables with high discriminative values. Furthermore, smaller ranges of backward motion of the trunk were found in both PD subtypes, which indicated an impaired trunk extension in PD. Earlier studies^57,58^ also observed a loss of the ability to extend the trunk in early stage of PD, which is in congruent with our findings indicating that the increased tension of trunk flexor muscle may contribute to the impaired trunk extension. And the reduced neuromuscular efficiency of trunk extension and impaired control of the trunk extensor force might also implicate the camptocormia in advanced PD patients^59^. As the flexibility and balance control of the trunk were impaired in early PD patients, the trunk motion during the process of sitting down and standing up was an “en bloc” transition instead of the fine and complex adjustments of postural transitions, which resulted in a shortened duration of postural transitions. This may provide an alternative explanation to the significantly shorter duration observed in our patients with PD compared with the HC.

Compared to TD patients, patients with PIGD are known to exhibit a faster disease progression with greater motor impairment, including a higher risk of falling, worse postural control, and a greater likelihood of experiencing FOG. Treatment for the PIGD subtype is also more challenging^21,60,61^. Proper identification of motor subtypes earlier in the disease course is important as it can help predict the progression of the disease and determine appropriate interventions to manage the progression of PD^62^. However, the conventional method for distinguishing PD motor subtypes involves resource-intensive physical examination by a movement disorders specialist^63^. In our wearable sensor-based gait study, we proposed some early gait metrics including bilateral arm peak velocity and turning average step duration could objectively differentiate between TD and PIGD subtypes, before the onset of clinically apparent gait impairment. Our results were partially consistent with the study conducted by Lazzaro et al.^40^, who analyzed different motion profiles of PD subtypes using wearable sensors in early stage of the disease, and found features related to gait metric and upper body movement resulted statistically significant to differentiate between TD and PIGD subtypes. Herman et al.^21^ and Wu et al.^64^ also attempted to quantify the objective gait metrics of these two PD subtypes using wearable device, and found the gait performance of patients with PIGD is worse than those with TD, such as the shorter strides, decreased stride regularity, and increased stride variability. However, PD subjects involved in these studies were not in early stage, and had an average disease duration of more than 5 years. Although significant gait features that can be used for subtype identification were analyzed in these studies, they did not further investigate the diagnostic values of these features in subtype differentiation. In addition, a recent study^63^ investigated a machine learning model utilizing full-body kinematic data during walking tasks to automatically and objectively identify PD motor subtypes. However, this study involved PD subjects in mid-to-advanced stages, with an average disease duration of more than 7 years. To objectively recognize the specific subtype as soon as possible, and to allow specific intervention at the earliest stage of PD, subjects with minimal motor abnormalities who were in the early phase of the disease should be included in the studies^65^. Overall, future researches on the subtype differentiation should focus on the large dataset, involving a wide number of subjects with PD at only a mild clinical stage of the disease.

For disease severity monitoring, we found the trunk sagittal peak velocity during the processes of sitting down and standing up reduced with the increase of disease severity in both TD and PIGD patients, which was in line with the result that trunk rotation deficits correlated to disease severity in another study by Verheyden et al.^66^. Additionally, the limitation of the backward swing of MAS arm and trunk transverse peak velocity showed the largest correlation with the severity of the disease as measured by the MDS-UPDRS motor scores in the TD and PIGD subtypes, respectively. Moreover, the arm swing velocity and parameters related turning showed significantly correlated with the disease severity in PIGD group. In the previous studies conducted by El-Gohary and Mancini^67,68^, they both underscored the value of specific turning metrics, such as duration and steps, as robust biomarkers for evaluating PD-related motor impairments. The MDS-UPDRS is currently among the most common and reliable protocols used to monitor the severity of the disease, although it remains a subjective and semiquantitative measure of motor symptoms. Our study demonstrated the wearable sensor-based gait analysis can provide objective assessments to monitor the disease severity in PD subtypes and may be helpful to implement and modify appropriate treatment plans for patients with different subtypes as needed.

This study faces limitations and future studies are needed. First, longitudinal study is needed to further support our findings and confirm the validity of gait parameters as early biomarkers of disease progression. Second, PD patients with special symptoms such as FOG and mild cognitive impairment have not been fully studied in the current study. Therefore, special studies focused on these symptoms of PD are needed to take the most advantage of the wearable sensor-based technology and obtain a more comprehensive understanding of the disease. Furthermore, future research should focus on exploring methods to integrate the most important variables into a single score for better clinical applicability.

Interestingly, based on our research, most gait biomarkers are relate to upper limbs, which facilitates the approach using fewer sensors, such as those in smartphones or smartwatches, for gait analysis, or leveraging smartphone-based machine vision with deep learning for gait assessment with the aim to reduce assessment costs, enhance convenience and suitability across various settings, and improve the continuity of evaluation over time. Additionally, AI-based gait evaluation systems hold great promise for managing PD and improving patient outcomes. Its advantages include higher diagnostic accuracy, continuous monitoring, and personalized therapeutic interventions^69^. Therefore, the current study also provides valuable gait biomarkers for the development of AI-based gait evaluation system, which may be helpful in improving PD diagnosis and treatment.

In conclusion, this pilot study provides a relatively comprehensive description of the objective gait alterations in early PD subtypes. Potential gait biomarkers for early diagnosis, subtype differentiation and disease severity monitoring have been further investigated. Although further studies on larger cohorts are warranted, the current results prove that the quantitative gait analysis based on wearable sensors may facilitate proper identification of motor subtypes early in the disease trajectory, enable monitor disease progression and guide the selection of appropriate interventions to treat and manage PD.

## Methods

### Participants

Forty-four patients with PD and 39 HCs participated. PD patients were recruited from the outpatient center of Ruijin Hospital affiliated with Shanghai Jiao Tong University School of Medicine and diagnosed by a movement disorders specialist (Dr. Shengdi Chen) according to the MDS clinical diagnostic criteria^70^. All PD patients participated in this study were in early stage (H-Y stage:1–2.5), and no camptocormia and marked festination or FOG was reported in PD participants. Of the total 39 HC, 28 were recruited from the community, 7 were spouses of the patients, 4 were other relatives (without family history) who volunteered to participate. All the HC were free from PD clinical manifestations. Exclusion criteria were age younger than 40 or older than 80, dementia, orthopedic comorbidities, any visual or vestibular impairment or physical limitations to perform the gait test. Written informed consent was obtained from each subject at the time of enrollment for clinical assessments. This study was approved by the ethic committee of Ruijin Hospital affiliated to Shanghai Jiao Tong University School of Medicine. The authors affirm that human research participant provided informed consent, for publication of the images in Figs. 1 and 2.

### Clinical evaluation and participant classification

The MDS-UPDRS^71^ and BBS^72^ scores were assessed in each PD subject and the modified H-Y stage^73,74^ was determined. PD patients were measured in an OFF-state (withdrawal of dopaminergic medication for 12 h) when they experienced an end-of-dose effect prior to intake of their next medication dose. Patients with PD were classified as TD, PIGD, or indeterminate based on sub-scores of MDS-UPDRS part II and III^4^. Briefly, the ratio of the average TD score and the average PIGD score was assessed. Patients with a ratio ≥1.15 were classified as TD and with a ratio ≤0.9 as PIGD^4^. If the scores were in between these values, they were considered as indeterminate and excluded from in this study. General cognition was assessed using MMSE in all participates^75^, subjects with an MMSE score <24 were excluded.

### Gait assessment

Gait measurement was instrumented by a wearable motion and gait quantitative evaluation system (MATRIX, MA11, GYENNO SCIENCE Co., Ltd., Shenzhen, China), previously validated^76–78^. As shown in Fig. 1, ten lightweight and inertial sensors with accelerometer and gyroscope were attached to each subject’s chest, lower back, and bilateral wrists, thighs, ankles and feet with elastic bands. Sampling rate is 100 Hz, and the measuring range of the accelerometer is ±8 g, that of the gyroscope is ±2000◦/s. They have the high resolution of 0.00024 g and 0.06◦/s, respectively. Each sensor collected spatial-temporal gait characteristics in real time during the TUG test and then transmitted the information to the host computer via a bluetooth link for further processing and storage.

Participants were instructed to stand up from a chair without armrests, walk at their usual pace on the ground comfortably for 5 m from the chair to the turning point, then walk back and sit. The ground was free from interference, and light was controlled. Before the formal tests, all subjects were encouraged to practice once to relieve the tension of walking with sensors and be familiar with the testing procedure. Then, each participant performed two consecutive walking trials and the average values of the gait parameters were investigated. Between trials subjects took a rest between 15 s and 30 s long, depending on whether and how long they needed to recover. Assessors were blinded to subject’s group at the time of assessment.

Given the asymmetry in PD, we further classified the selected lower and upper body parameters as MAS and LAS^26^. The MAS was determined based on the sum of the bradykinesia sub-scores of the MDS-UPDRS (Items 3.4, 3.5, and 3.6 for the upper body and Items 3.7 and 3.8 for the lower body). For control subjects, the average of both sides was used for analyzed. Sixty-four gait and postural-related features were obtained from ten body-fixed sensors of each participant during the TUG test. Among them, 31 were lower body-related gait features, 12 were trunk and lumbar-related gait features, 8 were upper body-related gait features, and 13 were postural transitions (including three parts: turning, sitting down, and standing up) related gait features. The description and corresponding formula of these features are detailed in Supplementary Table 2.

### Statistics

The statistical analyses were carried out with SPSS Statistics v.25 (IBM). Normality and homoscedasticity of data were tested with Shapiro-Wilk test and Levene’s test of equality of variances. Continuous variables were reported as the mean and standard deviation (SD) if they were normally distributed or as the median and quartile (Q)1~Q3 if they were not normally distributed.

Differences in demographic variables among the HC, TD and PIGD groups were compared using the analysis of variance (ANOVA) or Kruskal Wallis test for parametric and non-parametric test, respectively. Differences in clinical characteristics between the TD and PIGD groups were tested using two-sample *T* test or Mann–Whitney test for parametric and non-parametric test, respectively. Pearson’s Chi square test was used to compare categorical variables. Since plenty of gait variables were not normally distributed, the gait parameters among the control, TD, and PIGD groups were compared by using the Kruskal Wallis test. The Bonferroni correction was performed in multiple comparisons. In addition, the ROC analysis with the AUC value were used to evaluate and quantify the discriminatory ability of each gait variable in differential diagnosis between PD subtypes and the HC. Finally, the Spearman rank correlation test was performed to investigate the associations between MDS-UPDRS motor scores and selected gait features of PD subtypes (only those with a high discriminatory value and significant Kruskal–Wallis test). All hypotheses were non-directional and a statistically significant difference was indicated by a *p* value of <0.05.

### Reporting summary

Further information on research design is available in the Nature Research Reporting Summary linked to this article.

### Supplementary information

Supplementary Materials

Reporting summary

## Data Availability

The datasets generated and/or analyzed during the current study are available from the corresponding author upon reasonable request.

## Code availability

The standard software application has been used for data analysis and is mentioned in the methods section.
