# Supplementary material for: Wearable sensor-based quantitative gait analysis in Parkinson’s disease patients with different motor subtypes
Source: NPJ Digit Med. 2024 Jun 26;7:169. doi: 10.1038/s41746-024-01163-z (PMC11208588; doi:10.1038/s41746-024-01163-z)
Supplement: Supplementary file 1 — Supplementary Materials [file 41746_2024_1163_MOESM1_ESM.pdf]

Supplementary Materials for

Wearable sensor-based quantitative gait analysis in Parkinson's disease patients with different motor subtypes

Supplementary Table 1. Discriminative value of gait characteristics[a] for early diagnosis in PD subtypes and subtype differentiation.

Supplementary Table 2. Description of the gait features extracted from the TUG test using the wearable sensors.

Supplementary Table 1. Discriminative value of gait characteristics[a] for early diagnosis in PD subtypes and subtype differentiation.

Gait parameters	HC vs. TD			HC vs. PIGD			TD vs. PIGD		
	AUC	95% CI	*p*	AUC	95% CI	*p*	AUC	95% CI	*p*
**Lower body related parameters**									
MAS Swing (%)	0.713	0.571~0.856	0.005	0.704	0.554~0.855	0.012			
MAS Stance (%)	0.713	0.571~0.856	0.005	0.704	0.554~0.855	0.012			
MAS Shank Backward Swing Maximum (°)	0.81*	0.698~0.923	<0.001	0.779	0.654~0.903	0.001			
LAS Shank Backward Swing Maximum (°)	0.845*	0.747~0.943	<0.001	0.825*	0.714~0.935	<0.001			
MAS Peak Shank Angular Velocity (°/s)	0.705	0.576~0.833	0.007						
Shank Symbolic Symmetry Index (%)	0.683	0.537~0.83	0.017						
**Trunk and lumbar related parameters**									
Trunk Coronal Peak Velocity (°/s)				0.722	0.579~0.865	0.006			
Trunk Coronal RoM (°)	0.716	0.581~0.851	0.004	0.864*	0.769~0.96	<0.001			
Trunk Sagittal Peak Velocity (°/s)				0.731	0.577~0.886	0.004			
Trunk Transverse Peak Velocity (°/s)				0.702	0.546~0.857	0.013			
Lumbar Coronal RoM (°)	0.871*	0.785~0.956	<0.001	0.796	0.662~0.93	<0.001			
Lumbar Sagittal Peak Velocity (°/s)				0.703	0.558~0.849	0.013			
**Upper body related parameters**									
MAS Arm Peak Velocity (°/s)				0.835*	0.717~0.954	<0.001	0.792	0.656~0.927	<0.001
LAS Arm Peak Velocity (°/s)				0.715	0.567~0.863	0.008	0.757	0.599~0.914	0.001
MAS Arm Backward Swing Maximum (°)	0.871*	0.783~0.959	<0.001	0.879*	0.78~0.977	<0.001			
LAS Arm Backward Swing Maximum (°)				0.745	0.613~0.877	0.003			
Arm Velocity Asymmetry (%)	0.716	0.57~0.862	0.005						
Arm Symbolic Symmetry Index (%)	0.868*	0.757~0.979	<0.001	0.762	0.622~0.903	0.001			
**Postural transitions related parameters**									
Turning - Average Duration (s)	0.735	0.613~0.857	0.002	0.797	0.673~0.921	<0.001			

Turning - Peak Velocity (°/s)				0.802*	0.673~0.93	<0.001			
Turning - Average Angular Velocity (°/s)	0.74	0.619~0.862	0.002	0.799	0.676~0.922	<0.001			
Turning - Average Steps	0.749	0.622~0.877	0.001						
Turning - Average Step Duration (s)							0.736	0.587~0.886	0.002
SiSt - Trunk Sagittal Peak Velocity (°/s)	0.816*	0.712~0.92	<0.001	0.794	0.661~0.926	<0.001			
SiSt - Trunk Lean Backward Maximum (°)	0.705	0.569~0.84	0.007						
StSi - Duration (s)	0.786	0.667~0.905	<0.001						
StSi - Trunk Sagittal Peak Velocity (°/s)	0.709	0.58~0.839	0.006	0.731	0.594~0.867	0.004			
StSi- Trunk Lean Backward Maximum (°)	0.789	0.674~0.905	<0.001	0.744	0.61~0.878	0.002			

PD, Parkinson's disease; AUC, area under the curve; CI, confidence interval; HC, healthy controls; TD, tremor-dominant; PIGD, postural instability and gait disorder; MAS, more affected side; LAS, less affected side; RoM, range of motion; SiSt, sit to stand; StSi, stand to sit. a: gait variables with significant Kruskal Wallis test and the corresponding pair-wise comparison.*: discriminative parameters with an AUC >0.8.

Supplementary Table 2. Description of the gait features extracted from the TUG test using the wearable sensors.

Gait parameters	Components	M.U.	Description
			**Lower body related parameters**
Step Length	Gait	[cm]	The straight-line distance from the opposite heel to the ipsilateral heel in a Gait Cycle at the time when the unilateral heel strikes ground.<br>$steplen = \mathrm{len}(hs\_A - hs\_B)$<br>$steplen$: Step Length<br>$\mathrm{len}$: Indicates finding the length of two positions<br>hs_A: A side heel strike position<br>hs_B: B side heel strike position
Stride Velocity	Gait	[m/s]	The ratio of Stride Length to its corresponding ipsilateral Gait Cycle Duration.<br>$stide\_v = stide\_len / (t5 - t1)$<br>$stide\_v$: Stride Velocity<br>$stide\_len$: Stride length<br>$t1$: First ground contact moment in the gait cycle(s)<br>$t2$: Last ground contact moment in the gait cycle(s)
Stride Length	Gait	[cm]	The straight-line distance between a pair of successive ipsilateral heel-striking events.<br>$stride\_len = stepL + stepR$<br>$stride\_len$: Stride Length<br>$stepL$: Left step length<br>$stepR$: Right step length

Gait Cycle time	Gait	[s]	The time interval between two successive ipsilateral heel-striking events.<br>$GaitCycle = t5 - t1$<br>$GaitCycle$: Gait cycle time<br>$t1$: First ground contact moment in the gait cycle(s)<br>$t5$: Last ground contact moment in the gait cycle(s)
Cadence	Gait	[step/min]	The number of steps taken in a minute.<br>$Cadence = 60 / (t1 - t2)$<br>$Cadence$: Cadence<br>$t1$: A side ground contact moment in the gait cycle(s)<br>$t2$: B side ground contact moment in the gait cycle(s)
Double Support	Gait	[%]	The ratio of the double support phase duration to its corresponding ipsilateral Gait Cycle Duration.<br>$double\_s = 100 * (t2 - t1 + t4 - t3) / (t5 - t1)$<br>$double\_s$: Double support phase<br>$t1$: A side ground contact moment in the gait cycle(s)<br>$t2$: B side ground lift moment in the gait cycle(s)<br>$t3$: B side ground contact moment in the gait cycle(s)<br>$t4$: A side ground lift moment in the gait cycle(s)<br>$t5$: A side ground contact moment in the gait cycle(s)
Swing	Gait	[%]	The ratio of the period, during which the unilateral limb is unconnected with the ground, to its corresponding ipsilateral Gait Cycle.<br>$swing = 1 - st$<br>$swing$: Swing phase<br>st : Support phase

Stance	Gait	[%]	The ratio of the unilateral stance phase duration to its corresponding ipsilateral Gait Cycle Duration.<br>$st = 100 * (t4 - t1) / (t5 - t1)$<br>$st$: Support phase<br>$t1$: A side ground contact moment in the gait cycle(s)<br>$t4$: A side ground lift moment in the gait cycle(s)<br>$t5$: A side ground contact moment in the gait cycle(s)
Shank Forward Swing Maximum	Gait	[°]	The maximum angle value of the unilateral shank forward swings in a Gait Cycle.<br>$shank\_angle\_max = \max(shank\_pit(t1{:}t5))$<br>max : Find the maximum value<br>$shank\_angle\_max$: Shank Forward Swing Maximum<br>$shank\_pit$: Shank swing angle<br>$t1$: First ground contact moment in the gait cycle(s)<br>$t5$: Last ground contact moment in the gait cycle(s)
Shank Backward Swing Maximum	Gait	[°]	The maximum angle value of the unilateral shank backward swings in a Gait Cycle.<br>$shank\_angle\_min = \min(shank\_pit(t1{:}t5))$<br>min : Find the minimum value<br>$shank\_angle\_min$: Shank Backward Swing Maximum<br>$shank\_pit$: Shank swing angle<br>$t1$: First ground contact moment in the gait cycle(s)<br>$t5$: Last ground contact moment in the gait cycle(s)

Peak Shank Angular Velocity	Gait	[°/s]	The maximum angular velocity value of the unilateral shank swings in a Gait Cycle.<br>$shank\_angle\_vel\_max = \max(abs(shank\_gyro(t1:t5)))$<br>max : Find the maximum value<br>$shank\_angle\_vel\_max$: Peak Shank Angular Velocity<br>$shank\_gyro$: Shank swing angular velocity<br>$t1$: First ground contact moment in the gait cycle(s)<br>$t5$: Last ground contact moment in the gait cycle(s)
Stride Velocity Asymmetry	Gait	[%]	Difference in stride velocity between the left and right limb divided by the larger value.<br>$stride\_vel\_asym = 100 * abs(stride\_v\_L - stride\_v\_R) / \max([stride\_v\_L \ \ stride\_v\_R])$<br>abs : Get the absolute value<br>max : Find the maximum value<br>$stride\_vel\_asym$: Stride Velocity Asymmetry<br>$stride\_v\_L$: Left stride speed<br>$stride\_v\_R$: Right stride speed
Stride Length Asymmetry	Gait	[%]	Difference in stride length between the left and right limb divided by the larger value.<br>$stride\_len\_asym = 100 * \mathrm{abs}(stride\_lenL - stride\_lenR) / \max([stride\_lenL \ \ stride\_lenR])$<br>abs : Get the absolute value<br>max : Find the maximum value<br>$stride\_len\_asym$: Stride Length Asymmetry<br>$stride\_len\ L$: Left stride length<br>$stride\_len\ R$: Right stride length

Swing Asymmetry	Gait	[%]	Difference in swing phase between the left and right limb divided by the larger value.<br>$swing\_asym = 100 * \mathrm{abs}(swing\_L - swing\_R) / \max([swing\_L \;\; swing\_R])$<br>abs : Get the absolute value<br>max : Find the maximum value<br>$swing\_asym$: Swing Asymmetry<br>$swing\_L$: Left swing phase<br>$swing\_R$: Right swing phase
Stance Asymmetry	Gait	[%]	Difference in stance phase between the left and right limb divided by the larger value.<br>$st\_asym = 100 * \mathrm{abs}(st\_L - st\_R) / \max([st\_L \;\; st\_R])$<br>abs : Get the absolute value<br>max : Find the maximum value<br>st_asym : Stance Asymmetry<br>st_L : Left support phase<br>st_R : Right support phase
Shank RoM Asymmetry	Gait	[%]	Difference in range of motion between the left and right shank divided by the larger value.<br>$ShangkRomAsym = 100 * \mathrm{abs}(scope\_L - scope\_R) / \max([scope\_L \;\; scope\_R])$<br>abs : Get the absolute value<br>max : Find the maximum value<br>$ShangkRomAsym$: Shank RoM Asymmetry<br>$scope\_L$: Left shank angle range<br>$scope\_R$: Right shank angle range

Peak Shank Angular Velocity Asymmetry	Gait	[%]	Difference in peak angular velocity between the left and right shank divided by the larger value.<br>$PeakshankAngVelAsym = 100 * \text{abs}(w\_L - w\_R) / \max([w\_L\ w\_R])$<br>abs : Get the absolute value<br>max : Find the maximum value<br>$PeakshankAngVelAsym$: Peak Shank Angular Velocity Asymmetry<br>w_L : Peak angular velocity of left shank<br>w_R : Peak angular velocity of right shank
Shank Symbolic Symmetry Index	Gait	[%]	Shank Symbolic Symmetry Index is applied to describe the symmetry of shank movements. The smaller index refers to better symmetry.(1)
Mean Phase Difference	Gait	[%]	Can be applied to describe the coordination of the lower limbs. The lower the value means better symmetry. (2)
Phase Coordination Index	Gait	[%]	Can be applied to describe the coordination of the lower limbs. The lower the value means better coordination. (3)
			**Trunk and lumbar related parameters**
Trunk Coronal Peak Velocity	Gait	[°/s]	The coronal projection of the trunk maximum angular velocity in a Gait Cycle.<br>$TrunkCorPeakVel = \max(\text{abs}(front\_gyr\_x(t1:t5)))$<br>abs : Get the absolute value<br>max : Find the maximum value<br>$TrunkCorPeakVel$: Trunk Coronal Peak Velocity<br>$front\_gyr\_x$: The output of the x-axis of the chest gyroscope(°/s)<br>$t1$ and $t5$ are the start and end time of each gait cycle respectively

Trunk Coronal RoM	Gait	[°]	The range of motion of the trunk in left-right plane.<br>$TrunkCorRom = TrunkCorMax - TrunkCorMin$<br>$TrunkCorMax$: Maximum value of trunk right tilt angle<br>$TrunkCorMin$: Maximum value of trunk left tilt angle<br>$TrunkCorRom$: Trunk Coronal RoM
Trunk Sagittal Peak Velocity	Gait	[°/s]	The sagittal projection of the trunk maximum angular velocity in a Gait Cycle.<br>$TrunkSagPeakVel = \max(\text{abs}(front\_gyr\_y(t1:t5)))$<br>abs : Get the absolute value<br>max : Find the maximum value<br>$TrunkSagPeakVel$: Trunk Sagittal Peak Velocity<br>$front\_gyr\_y$: The output of the y-axis of the chest gyroscope<br>$t1$ and $t5$ are the start and end time of each gait cycle respectively
Trunk Sagittal RoM	Gait	[°]	The range of motion of the trunk in anterior-posterior plane.<br>$TrunkSagRom = TrunkSagMax - TrunkSagMin$<br>$TrunkSagMax$: Maximum trunk backward tilt angle<br>$TrunkSagMin$: Maximum trunk forward tilt angle<br>$TrunkSagRom$: Trunk Sagittal RoM
Trunk Transverse Peak Velocity	Gait	[°/s]	The transverse projection of the trunk maximum angular velocity in a Gait Cycle.<br>$TrunkTraPeakVel = \max(\text{abs}(front\_gyr\_z(t1:t5)))$<br>abs : Get the absolute value<br>max : Find the maximum value<br>$TrunkTraPeakVel$: Trunk Transverse Peak Velocity<br>$front\_gyr\_z$: The output of the z-axis of the chest gyroscope(°/s),<br>$t1$ and $t5$ are the start and end time of each gait cycle respectively

Trunk Transverse RoM	Gait	[°]	The range of motion of the trunk in transverse plane.<br>$TrunkTraRom = TrunkTraMax - TrunkTraMin$<br>$TrunkTraMax$: Maximum angle of trunk right rotation<br>$TrunkTraMin$: Maximum angle of trunk left rotation<br>$TrunkTraRom$: Trunk Transverse RoM
Lumbar Coronal Peak Velocity	Gait	[°/s]	The coronal projection of the lumbar maximum angular velocity in a Gait Cycle.<br>$LumbarCorPeakVel = \max(\mathrm{abs}(waist\_gry\_x(t1:t5)))$<br>abs : Get the absolute value<br>max : Find the maximum value<br>$LumbarCorPeakVel$: Lumbar Coronal Peak Velocity<br>$waist\_gry\_x$: The output of the x-axis of the lumbar gyroscope(°/s)<br>$t1$ and $t5$ are the start and end time of each gait cycle respectively
Lumbar Coronal RoM	Gait	[°]	The range of motion of the waist in left-right plane.<br>$LumbarCorRom = LumbarCorMax - LumbarCorMin$<br>$LumbarCorMax$: Maximum value of lumbar right tilt angle<br>$LumbarCorMin$: Maximum value of lumbar left tilt angle<br>$LumbarCorRom$: Lumbar Coronal RoM
Lumbar Sagittal Peak Velocity	Gait	[°/s]	The sagittal projection of the lumbar maximum angular velocity in a Gait Cycle.<br>$LumbarSagPeakVel = \max(\mathrm{abs}(waist\_gyr\_y(t1:t5)))$<br>abs : Get the absolute value<br>max : Find the maximum value<br>$LumbarSagPeakVel$: Lumbar Sagittal Peak Velocity<br>$waist\_gyr\_y$: The output of the y-axis of the lumbar gyroscope(°/s)<br>$t1$ and $t5$ are the start and end time of each gait cycle respectively

Lumbar Sagittal RoM	Gait	[°]	The range of motion of the waist in anterior-posterior plane.<br>$LumbarSagRom = LumbarSagMax - LumbarSagMin$<br>$LumbarSagMax$: Maximum lumbar backward tilt angle<br>$LumbarSagMin$: Maximum lumbar forward tilt angle<br>$LumbarSagRom$: Lumbar Sagittal RoM
Lumbar Transverse Peak Velocity	Gait	[°/s]	The transverse projection of the lumbar maximum angular velocity in a Gait Cycle.<br>$LumbarTraPeakVel = \max(\text{abs}(waist\_gyr\_z(t1:t5)))$<br>abs : Get the absolute value<br>max : Find the maximum value<br>$LumbarTraPeakVel$: Lumbar Transverse Peak Velocity<br>$waist\_gyr\_z$: The output of the z-axis of the lumbar gyroscope(°/s)<br>$t1$ and $t5$ are the start and end time of each gait cycle respectively
Lumbar Transverse RoM	Gait	[°]	The range of motion of the waist in transverse plane.<br>$LumbarTraRom = LumbarTraMax - LumbarTraMin$<br>$LumbarTraMax$: Maximum angle of lumbar right rotation<br>$LumbarTraMin$: Maximum angle of lumbar left rotation<br>$LumbarTraRom$: Lumbar Transverse RoM
			**Upper body related parameters**
Arm Peak Velocity	Gait	[°/s]	The sagittal projection of the unilateral-arm maximum angular velocity in each gait cycle.<br>$ArmPeakVel = \max(arm\_gyr\_norm(t1:t5))$<br>max : Find the maximum value<br>$ArmPeakVel$: Arm Peak Velocity<br>arm_gyr_norm : arm angular velocity<br>$t1$ and $t5$ are the start and end time of each gait cycle respectively

Arm Forward Swing Maximum	Gait	[°]	The sagittal projection of the unilateral-arm maximum angle forward in each gait cycle.<br>$ArmRomMax = \max(arm\_pit(t1{:}t5))$<br>$\max$: Find the maximum value<br>$ArmRomMax$: Arm Forward Swing Maximum<br>arm_pit : Pitch angle of arm(°),<br>$t1$ and $t5$ are the start and end time of each gait cycle respectively
Arm Backward Swing Maximum	Gait	[°]	The sagittal projection of the unilateral-arm maximum angle backward in each gait cycle.<br>$ArmRomMax = \min(arm\_pit(t1{:}t5))$<br>$\min$: Find the minimum value<br>$ArmRomMax$: Arm Backward Swing Maximum<br>arm_pit : Pitch angle of arm(°)<br>$t1$ and $t5$ are the start and end time of each gait cycle respectively
Arm Velocity Asymmetry	Gait	[%]	Difference in peak arm swing velocity between the left and right arm divided by the larger value.<br>$ArmSwingVelAsym = \text{abs}(ArmPeak\_L - ArmPeak\_R) / \max([ArmPeak\_L\ ArmPeak\_R])$<br>$\text{abs}$: Get the absolute value<br>$\max$: Find the maximum value<br>$ArmSwingVelAsym$: Arm Velocity Asymmetry<br>$ArmPeak\_L$: Left arm swing maximum angle<br>$ArmPeak\_R$: Right arm swing maximum angle
Arm Symbolic Symmetry Index	Gait	[%]	Arm Symbolic Symmetry Index is applied to describe the symmetry of arm swings. The smaller index refers to better symmetry. (1)

Postural transitions related parameters

Turning - Average Duration	Turning	[s]	The duration of the turning process.<br>$Average\_dur = \text{mean}([turn\_dur1 \;\; turn\_dur2])$<br>mean : Calculate the average<br>$Average\_dur$: Turning - Average Duration<br>$turn\_dur1$: First turn time<br>$turn\_dur2$: Second turn time
Turning - Peak Velocity	Turning	[°/s]	The maximum achieved angular velocity of trunk rotation in the transverse plane during 180-degree turns.<br>$PeakTurningAngVel = \max(\text{abs}(front\_gyr\_z(ts:te)))$<br>abs : Get the absolute value<br>max : Find the maximum value<br>$PeakTurningAngVel$: Turning - Peak Velocity<br>$front\_gyr\_z$: The output of the z-axis of the chest gyroscope(°/s)<br>ts : Turn start time<br>te : Turn end time
Turning - Average Step Duration	Turning	[s]	Turning Duration divided by Average Steps.<br>$StepTime = turn\_dur / steps$<br>$StepTime$: Turning - Average Step Duration<br>$turn\_dur$: Turn duration<br>$steps$: The average number of steps in the turning process

Turning - Average Angular Velocity	Turning	[°/s]	The angle of turning divided by the time during which the subject can complete the turning.<br>$MeanTurningAngVelocity = turn\_angle / (te - ts)$<br>$MeanTurningAngVelocity$: Turning - Average Angular Velocity<br>$turn\_angle$: The angle of turning<br>ts : Turn start time<br>te : Turn end time
Turning - Average Steps	Turning	[step]	The count of the steps during turning.<br>$Average\_steps = \text{mean}([turn\_step1\ \ turn\_step2])$<br>mean : Calculate the average<br>$Average\_steps$: Turning - Average Steps<br>$turn\_step1$: First turn step count<br>$turn\_step2$: Second turn step count
SiSt - Duration	Sit-to-Stand	[s]	The duration of the sit-to-stand process.<br>$sist\_dur = sist\_e - sist\_s$<br>$sist\_dur$: SiSt - Average Duration<br>$sist\_s$: Stand up start time<br>$sist\_e$: Stand up end time
SiSt - Trunk Sagittal Peak Velocity	Sit-to-Stand	[°/s]	The maximum angular velocity of trunk in anterior-posterior plane during the sit-to-stand process.<br>$TrunkSagPeakVel = \max(\text{abs}(front\_gyr\_y(sist\_s{:}sist\_e)))$<br>abs : Get the absolute value<br>max : Find the maximum value<br>$TrunkSagPeakVel$: SiSt - Trunk Sagittal Peak Velocity<br>$front\_gyr\_y$: The output of the y-axis of the chest gyroscope(°/s)<br>$sist\_s$: Stand up start time<br>$sist\_e$: Stand up end time

SiSt - Trunk Lean Backward Maximum	Sit-to-Stand	[°]	The maximum backward-angle during the sit-to-stand process.<br>$TrunkSagMax = \max(front\_pit(sist\_s : sist\_e))$<br>$\max$: Find the maximum value<br>$TrunkSagMax$: SiSt - Trunk Lean Backward Maximum<br>$front\_pit$: Pitch of chest node(°)<br>$sist\_s$: Stand up start time<br>$sist\_e$: Stand up end time
SiSt - Trunk Lean Forward Maximum	Sit-to-Stand	[°]	The maximum forward-angle during the sit-to-stand process.<br>$TrunkSagMax = \min(front\_pit(sist\_s : sist\_e))$<br>$\min$: Find the minimum value<br>$TrunkSagMax$: SiSt - Trunk Lean Forward Maximum<br>$front\_pit$: Pitch angle of chest node(°)<br>$sist\_s$: Stand up start time<br>$sist\_e$: Stand up end time
StSi - Duration	Stand-to-Sit	[s]	The duration of the stand-to-sit process.<br>$stsi\_dur = stsi\_e - stsi\_s$<br>$stsi\_dur$: SiSt - Average Duration<br>$stsi\_s$: Sit down start time<br>$stsi\_e$: Sit down end time

StSi - Trunk Sagittal Peak Velocity	Stand-to-Sit	[°/s]	The maximum angular velocity of trunk in anterior-posterior plane during the stand-to-sit process.<br>$TrunkSagiPeakVel = \max(\mathrm{abs}(front\_gyr\_y(stsi\_s{:}stsi\_e)))$<br>max : Find the maximum value<br>$TrunkSagiPeakVel$: StSi - Trunk Sagittal Peak Velocity<br>$front\_gyr\_y$: The output of the y-axis of the chest gyroscope(°/s)<br>$stsi\_s$: Sit down start time<br>$stsi\_e$: Sit down end time
StSi - Trunk Lean Backward Maximum	Stand-to-Sit	[°]	The maximum backward-angle during the stand-to-sit process.<br>$TrunkSagRomMax = \max(front\_pit(stsi\_s{:}stsi\_e))$<br>max : Find the maximum value<br>$TrunkSagRomMax$: StSi - Trunk Lean Backward Maximum<br>$front\_pit$: Pitch angle of chest node(°),<br>$stsi\_s$: Sit down start time<br>$stsi\_e$: Sit down end time
StSi - Trunk Lean Forward Maximum	Stand-to-Sit	[°]	The maximum forward-angle during the stand-to-sit process.<br>$TrunkSagRomMin = \min(front\_pit(stsi\_s{:}stsi\_e))$<br>min : Find the minimum value<br>$TrunkSagRomMin$: StSi - Trunk Lean Forward Maximum<br>$front\_pit$: Pitch angle of chest node(°)<br>$stsi\_s$: Sit down start time<br>$stsi\_e$: Sit down end time

cm, centimeter; m, meter; s, second; min, minute; %, percent; °, degree.
RoM, range of motion; SiSt, sit to stand; StSi, stand to sit.

Supplementary References

1. Sant'Anna A, Salarian A, Wickström N. A new measure of movement symmetry in early Parkinson's disease patients using symbolic processing of inertial sensor data. *IEEE Trans Biomed Eng.* Jul 2011;58(7):2127-2135. doi:10.1109/tbme.2011.2149521
2. Zivotofsky AZ, Gruendlinger L, Hausdorff JM. Modality-specific communication enabling gait synchronization during over-ground side-by-side walking. *Hum Mov Sci.* Oct 2012;31(5):1268-1285. doi:10.1016/j.humov.2012.01.003
3. Plotnik M, Giladi N, Hausdorff JM. A new measure for quantifying the bilateral coordination of human gait: effects of aging and Parkinson's disease. *Exp Brain Res.* Aug 2007;181(4):561-570. doi:10.1007/s00221-007-0955-7
