# Supplementary material for: Wearable sensor-based quantitative gait analysis in Parkinson’s disease patients with different motor subtypes
Source: NPJ Digit Med. 2024 Jun 26;7:169. doi: 10.1038/s41746-024-01163-z (PMC11208588; doi:10.1038/s41746-024-01163-z)
Supplement: Supplementary file 2 — Reporting summary [file 41746_2024_1163_MOESM2_ESM.pdf]

nature portfolio

Corresponding author(s): Pei Huang & Yuyan Tan

Last updated by author(s): May 13, 2024

Reporting Summary

Nature Portfolio wishes to improve the reproducibility of the work that we publish. This form provides structure for consistency and transparency in reporting. For further information on Nature Portfolio policies, see our Editorial Policies and the Editorial Policy Checklist.

Statistics

For all statistical analyses, confirm that the following items are present in the figure legend, table legend, main text, or Methods section.

n/a	Confirmed	
☐	☒	The exact sample size (*n*) for each experimental group/condition, given as a discrete number and unit of measurement
☐	☒	A statement on whether measurements were taken from distinct samples or whether the same sample was measured repeatedly
☐	☒	The statistical test(s) used AND whether they are one- or two-sided<br>*Only common tests should be described solely by name; describe more complex techniques in the Methods section.*
☐	☒	A description of all covariates tested
☐	☒	A description of any assumptions or corrections, such as tests of normality and adjustment for multiple comparisons
☐	☒	A full description of the statistical parameters including central tendency (e.g. means) or other basic estimates (e.g. regression coefficient) AND variation (e.g. standard deviation) or associated estimates of uncertainty (e.g. confidence intervals)
☐	☒	For null hypothesis testing, the test statistic (e.g. *F*, *t*, *r*) with confidence intervals, effect sizes, degrees of freedom and *P* value noted<br>*Give P values as exact values whenever suitable.*
☒	☐	For Bayesian analysis, information on the choice of priors and Markov chain Monte Carlo settings
☒	☐	For hierarchical and complex designs, identification of the appropriate level for tests and full reporting of outcomes
☐	☒	Estimates of effect sizes (e.g. Cohen's *d*, Pearson's *r*), indicating how they were calculated

Our web collection on statistics for biologists contains articles on many of the points above.

Software and code

Policy information about availability of computer code

Data collection	Basic information and clinical assessment of each participant was collected at the time of recruitment, and the gait metrics were obtained from a wearable motion and gait quantitative evaluation system (MATRIX, MA11, GYENNO SCIENCE Co., Ltd., Shenzhen, China). The device has been certified by the Food and Drug Administration (FDA), European Conformity (CE) Medical, and National Medical Products Administration (NMPA).
Data analysis	SPSS v.25 IBM was used for statistical analysis.

For manuscripts utilizing custom algorithms or software that are central to the research but not yet described in published literature, software must be made available to editors and reviewers. We strongly encourage code deposition in a community repository (e.g. GitHub). See the Nature Portfolio guidelines for submitting code & software for further information.

Data

Policy information about availability of data

All manuscripts must include a data availability statement. This statement should provide the following information, where applicable:

- Accession codes, unique identifiers, or web links for publicly available datasets
- A description of any restrictions on data availability
- For clinical datasets or third party data, please ensure that the statement adheres to our policy

The datasets generated and/or analyzed during the current study are available from the corresponding author on reasonable request.

Research involving human participants, their data, or biological material

Policy information about studies with human participants or human data. See also policy information about sex, gender (identity/presentation), and sexual orientation and race, ethnicity and racism.

Reporting on sex and gender	Forty-four patients with Parkinson's disease (PD) and 39 healthy controls (HC) were involved in the study. PD patients were classified as 24 tremor-dominant (TD) and 20 postural instability and gait difficulty (PIGD) subtypes. The percentage of male subjects in HC, TD and PIGD groups were 56.4%, 62.5% and 55%, and the gender was matched among TD, PIGD and the HC. groups. The effect of gender on the gait biomarkers was not investigated in the present study, since this is beyond the scope of the current study.
Reporting on race, ethnicity, or other socially relevant groupings	All participants in this study were native Mandarin speakers from China.
Population characteristics	Forty-four patients with Parkinson's disease (PD) and 39 healthy controls (HC) were involved in the study. PD patients were classified as 24 tremor-dominant (TD) and 20 postural instability and gait difficulty (PIGD) subtypes. The mean or median of the age for HC, TD and PIGD groups were 66, 61.63, 62.05; the percentage of male subjects in HC, TD and PIGD groups were 56.4%, 62.5% and 55%; the mean of the height for HC, TD and PIGD groups were 163.77,166.83 and 166 cm; the mean or median of the weight for HC, TD and PIGD groups were 66, 67.65 and 68.23kg. The mean or median of the BMI for HC, TD and PIGD groups were 25.2, 24.23 and 24.54kg/m2.
Recruitment	PD patients were recruited from the outpatient center of Ruijin Hospital affiliated with Shanghai Jiao Tong University School of Medicine and diagnosed by a movement disorders specialist according to the MDS clinical diagnostic criteria. And all the participants followed the strict criteria for inclusion and exclusion. Of the total 39 HC, 28 were recruited from the community, 7 were spouses of the patients, 4 were other relatives who volunteered to participate. All the HC were free from PD clinical manifestations.
Ethics oversight	The study was approved by the ethic committee of Ruijin Hospital affiliated to Shanghai Jiao Tong University School of Medicine. Written informed consent was obtained from each participant.

Note that full information on the approval of the study protocol must also be provided in the manuscript.

Field-specific reporting

Please select the one below that is the best fit for your research. If you are not sure, read the appropriate sections before making your selection.

☒ Life sciences ☐ Behavioural & social sciences ☐ Ecological, evolutionary & environmental sciences

For a reference copy of the document with all sections, see nature.com/documents/nr-reporting-summary-flat.pdf

Life sciences study design

All studies must disclose on these points even when the disclosure is negative.

Sample size	Forty-four patients with PD and 39 healthy controls, were involved in the study. The sample size selection in this study was based on other published clinical studies of which the research population, research type and statistical method are consistent with ours.
Data exclusions	We excluded the data of indeterminate subtype of PD in the study based on the purpose of the research, which has been stated in the manuscript.
Replication	As a clinical study, sufficient samples and scientific methods could guarantee the repeatability of the results. The parameters of the wearable device were described in the Methods and detailed description and mathematical definition for each gait feature were provided in Supplementary Table1, which may facilitate the gait features interpretation and results replication.
Randomization	This pilot study is a cross-sectional study, without intervention. Strict inclusion and exclusion criteria of case group and control group are important and the randomization is not available in this study.
Blinding	The gait metrics of all participants were collected by assessors blinded to subject's group at the time of assessment.

Behavioural & social sciences study design

All studies must disclose on these points even when the disclosure is negative.

Study description	
Research sample	

Sampling strategy

Data collection

Timing

Data exclusions

Non-participation

Randomization

Ecological, evolutionary & environmental sciences study design

All studies must disclose on these points even when the disclosure is negative.

Study description

Research sample

Sampling strategy

Data collection

Timing and spatial scale

Data exclusions

Reproducibility

Randomization

Blinding

Did the study involve field work? ☐ Yes ☐ No

Field work, collection and transport

Field conditions

Location

Access & import/export

Disturbance

Reporting for specific materials, systems and methods

We require information from authors about some types of materials, experimental systems and methods used in many studies. Here, indicate whether each material, system or method listed is relevant to your study. If you are not sure if a list item applies to your research, read the appropriate section before selecting a response.

Materials & experimental systems

n/a	Involved in the study
☒	☐ Antibodies
☒	☐ Eukaryotic cell lines
☒	☐ Palaeontology and archaeology
☒	☐ Animals and other organisms
☐	☒ Clinical data
☒	☐ Dual use research of concern
☒	☐ Plants

Methods

n/a	Involved in the study
☒	☐ ChIP-seq
☒	☐ Flow cytometry
☒	☐ MRI-based neuroimaging

Antibodies

Antibodies used

Validation

Eukaryotic cell lines

Policy information about cell lines and Sex and Gender in Research

Cell line source(s)

Authentication

Mycoplasma contamination

Commonly misidentified lines
(See ICLAC register)

Palaeontology and Archaeology

Specimen provenance

Specimen deposition

Dating methods

☐ Tick this box to confirm that the raw and calibrated dates are available in the paper or in Supplementary Information.

Ethics oversight

Note that full information on the approval of the study protocol must also be provided in the manuscript.

Animals and other research organisms

Policy information about studies involving animals; ARRIVE guidelines recommended for reporting animal research, and Sex and Gender in Research

Laboratory animals

Wild animals

Reporting on sex

Field-collected samples

Ethics oversight

Note that full information on the approval of the study protocol must also be provided in the manuscript.

Clinical data

Policy information about clinical studies

All manuscripts should comply with the ICMJE guidelines for publication of clinical research and a completed CONSORT checklist must be included with all submissions.

Clinical trial registration	This is not a clinical trial study.
Study protocol	The study design was approved by the ethic committee of Ruijin Hospital affiliated to Shanghai Jiao Tong University School of Medicine.
Data collection	Basic information was obtained from each subject at the time of enrollment for clinical assessment. The MDS-UPDRS and BBS scores were assessed in each PD subject and the modified H-Y stage was determined. PD patients were measured in an OFF-state when they experienced an end-of- dose effect prior to intake of their next medication dose. General cognition was assessed using MMSE in all participants. Gait measurement was instrumented by a wearable motion and gait quantitative evaluation system (MATRIX, MA11, GYENNO SCIENCE Co., Ltd., Shenzhen, China). Ten lightweight and inertial sensors with accelerometer and gyroscope were attached to each subject's chest, lower back, and bilateral wrists, thighs, ankles and feet with elastic bands. Sampling rate is 100Hz, and the measuring range of the accelerometer is ±8 g, that of the gyroscope is ±2000∘/s. They have the high resolution of 0.00024g and 0.06∘/s respectively. Each sensor collected spatial-temporal gait characteristics in real time during the TUG test and then transmitted the information to the host computer via a bluetooth link for further processing and storage.
Outcomes	There is no expectation of primary or secondary treatment outcome, since this is not a clinical trial but a cross-sectional study for objective gait biomarkers in early PD subtypes based on the wearable sensors.

Dual use research of concern

Policy information about dual use research of concern

Hazards

Could the accidental, deliberate or reckless misuse of agents or technologies generated in the work, or the application of information presented in the manuscript, pose a threat to:

No	Yes	
☐	☐	Public health
☐	☐	National security
☐	☐	Crops and/or livestock
☐	☐	Ecosystems
☐	☐	Any other significant area

Experiments of concern

Does the work involve any of these experiments of concern:

No	Yes	
☐	☐	Demonstrate how to render a vaccine ineffective
☐	☐	Confer resistance to therapeutically useful antibiotics or antiviral agents
☐	☐	Enhance the virulence of a pathogen or render a nonpathogen virulent
☐	☐	Increase transmissibility of a pathogen
☐	☐	Alter the host range of a pathogen
☐	☐	Enable evasion of diagnostic/detection modalities
☐	☐	Enable the weaponization of a biological agent or toxin
☐	☐	Any other potentially harmful combination of experiments and agents

Plants

Seed stocks | N/A

Novel plant genotypes | N/A

Authentication | N/A

ChIP-seq

Data deposition

☐ Confirm that both raw and final processed data have been deposited in a public database such as GEO.

☐ Confirm that you have deposited or provided access to graph files (e.g. BED files) for the called peaks.

Data access links
May remain private before publication.

Files in database submission

Genome browser session
(e.g. UCSC)

Methodology

Replicates

Sequencing depth

Antibodies

Peak calling parameters

Data quality

Software

Flow Cytometry

Plots

Confirm that:

☐ The axis labels state the marker and fluorochrome used (e.g. CD4-FITC).

☐ The axis scales are clearly visible. Include numbers along axes only for bottom left plot of group (a 'group' is an analysis of identical markers).

☐ All plots are contour plots with outliers or pseudocolor plots.

☐ A numerical value for number of cells or percentage (with statistics) is provided.

Methodology

Sample preparation

Instrument

Software

Cell population abundance

Gating strategy

☐ Tick this box to confirm that a figure exemplifying the gating strategy is provided in the Supplementary Information.

Magnetic resonance imaging

Experimental design

Design type

Design specifications

Behavioral performance measures

Acquisition

Imaging type(s)

Field strength

Sequence & imaging parameters

Area of acquisition

Diffusion MRI ☐ Used ☐ Not used

Preprocessing

Preprocessing software

Normalization

Normalization template

Noise and artifact removal

Volume censoring

Statistical modeling & inference

Model type and settings

Effect(s) tested

Specify type of analysis: ☐ Whole brain ☐ ROI-based ☐ Both

Statistic type for inference

(See Eklund et al. 2016)

Correction

Models & analysis

n/a	Involved in the study
☐	☐ Functional and/or effective connectivity
☐	☐ Graph analysis
☐	☐ Multivariate modeling or predictive analysis

Functional and/or effective connectivity

Graph analysis

Multivariate modeling and predictive analysis
